# Supplementary material for: Effects of implementation of a care bundle on rates of necrotising enterocolitis and own mother’s milk feeding in the East Midlands: protocol for a mixed methods impact and process evaluation study
Source: BMJ Open. 2024 May 30;14(5):e078633. doi: 10.1136/bmjopen-2023-078633 (PMC11141194; doi:10.1136/bmjopen-2023-078633)
Supplement: Author's manuscript [file bmjopen-2023-078633.draft_revisions.pdf]

BMJ Open

BMJ Open is committed to open peer review. As part of this commitment we make the peer review history of every article we publish publicly available.

When an article is published we post the peer reviewers' comments and the authors' responses online. We also post the versions of the paper that were used during peer review. These are the versions that the peer review comments apply to.

The versions of the paper that follow are the versions that were submitted during the peer review process. They are not the versions of record or the final published versions. They should not be cited or distributed as the published version of this manuscript.

BMJ Open is an open access journal and the full, final, typeset and author-corrected version of record of the manuscript is available on our site with no access controls, subscription charges or pay-per-view fees (http://bmjopen.bmj.com).

If you have any questions on BMJ Open's open peer review process please email info.bmjopen@bmj.com

Effects of implementation of a care bundle on rates of necrotising enterocolitis and own mother's milk feeding in the East Midlands: protocol for a mixed methods impact and process evaluation study

Journal:	*BMJ Open*
Manuscript ID	bmjopen-2023-078633
Article Type:	Protocol
Date Submitted by the Author:	08-Aug-2023

Complete List of Authors:	Abramson, Janine; University of Nottingham<br>Szatkowski, Lisa; University of Nottingham, School of Medicine<br>Bains, Manpreet; University of Nottingham, Epidemiology and Public Health<br>Orton, Elizabeth; University of Nottingham<br>Budge, Helen; University of Nottingham; University of Nottingham<br>Spruce, Marie; NEC UK<br>Ojha, Shalini; University of Nottingham; University Hospitals of Derby and Burton NHS Foundation Trust, School of Medicine

Keywords:	NUTRITION & DIETETICS, NEONATOLOGY, Nutritional support < GASTROENTEROLOGY

SCHOLARONE™ Manuscripts

Effects of implementation of a care bundle on rates of necrotising enterocolitis and own mother's milk feeding in the East Midlands: protocol for a mixed methods impact and process evaluation study

Janine Abramson[1,2], Lisa Szatkowski[1,2], Manpreet Bains[1], Elizabeth Orton[1], Helen Budge[1,2], Marie Spruce[3], Shalini Ojha[1,2,4]

[1] Lifespan and Population Health, School of Medicine, University of Nottingham, UK

[2] Centre for Perinatal Research, School of Medicine, University of Nottingham, UK

[3] NEC UK, 42 Long Hill Rise, Annesley, Nottingham, NG17 9FG

[4] Neonatal Unit, University Hospitals of Derby and Burton NHS Trust, Derby, UK

Corresponding author: Professor Shalini Ojha, Lifespan and Population Health, School of Medicine, University of Nottingham. Room 4117, Medical School Building, Royal Derby Hospital, Derby DE22 3NE; email: shalini.ojha@nottingham.ac.uk

Orchid ID: https://orcid.org/0000-0001-5668-4227

Key words: infant, preterm; breastfeeding; necrotising enterocolitis

For peer review only

Abstract

300 words

Introduction

Prevention of necrotising enterocolitis (NEC) is vital for achieving the NHS goal to improve neonatal outcomes. Feeding own mother's milk helps prevent NEC. Rates of own mother's milk feeding in the East Midlands are lower than the national average and the incidence of NEC is higher. The East Midlands Neonatal Operational Delivery Network (EMNODN) has created a care bundle to improve these in babies born at <32 weeks' gestation, the group at the highest risk of NEC. The bundle was introduced in September 2022 and embedded by December 2022. We will evaluate its effectiveness and conduct a process evaluation to understand barriers and facilitators to implementation.

Methods

In Workstream 1, is a retrospective cohort study using data from the National Neonatal Research Database (NNRD). We will identify infants receiving any own mother's milk on day 14 and at discharge, and cases of severe NEC, aggregate outcomes by month of birth, and use interrupted time series analysis to estimate an incidence rate ratio (IRR) for changes after the care bundle was embedded, relative to pre-implementation. We will similarly model data from all other NNRD units and assess whether there are any concurrent changes to exclude confounding due to other events.

In Workstream 2, we will apply the RE-AIM framework to conduct a mixed methods process evaluation in the 11 EMNODN units. We will triangulate data from a variety of sources, including questionnaires and semi-structured interviews with parents and healthcare professionals, and data collected directly from patient records.

Ethics

Approved by the South-East Scotland Research Ethics Committee01.

For peer review only - http://bmjopen.bmj.com/site/about/guidelines.xhtml

Dissemination

Findings will be disseminated via scientific journals and conferences, to neonatal service commissioners and through public-facing infographics.

Implications

Evidence on the effectiveness of the care bundle will be used to support its maintenance and adoption beyond the East Midlands.

Study registration: ClinicalTrials.gov NCT05934123

For peer review only

For peer review only - http://bmjopen.bmj.com/site/about/guidelines.xhtml

Article Summary

Strengths and limitations of this study

- Use of data from the National Neonatal Research Database (NNRD) allows comparison of all infants admitted to neonatal units in the East Midlands with a control group of all infants admitted to units elsewhere in England and Wales.
- Interrupted time series analysis will allow any changes in own mother's milk feeding and the incidence of NEC related to implementation of the care bundle to be identified over and above any underlying changing trends.
- The parallel mixed methods process evaluation will allow us to unpick the 'how' and 'why' underpinning the results of the interrupted time series analysis.
- Analysis of routine data will allow all eligible infants to participate without risk of under-recruitment from underserved groups. In the process evaluation, we will purposively seek to include parents from underserved groups who traditionally hesitate to participate in research.
- NEC is a rare occurrence and so statistical power to detect small changes in incidence related to implementation of the care bundle may be limited.

For peer review only

Introduction

Necrotising enterocolitis (NEC) is a serious, life-threatening disease in preterm infants[1]. An estimated 12% of infants born weighing <1500g develop NEC and 30% of these die[2]. Survivors have increased risk of adverse neurodevelopmental outcomes, including cerebral palsy and cognitive impairment[3]. Encouraging and supporting own mother's milk feeding prevents NEC and has several additional advantages, including improved long-term neurodevelopment and parent-infant bonding[4]. Health economic analysis estimates that if all premature infants were to be fed own mother's milk, the total lifetime cost saving to the NHS would be £46.7 million (£30.1 million in the first year of life) and there would be 238 fewer deaths per year with a gain of £153.4 million in lifetime productivity[5].

Neonatal critical care services in England are commissioned by NHS England and managed within 11 Neonatal Operational Delivery Networks (ODNs), to ensure that babies and their families receive high quality, equitable and accessible care. The rate of NEC in the East Midlands Neonatal Operational Delivery Network (EMNODN[6]) has remained above the national average since 2017, when this measure was added to the National Neonatal Audit Programme, NNAP[7]. Additionally, there are wide variations between the 11 units (across 7 NHS Trusts) that comprise the EMNODN. Rates of any breastmilk feeding at discharge from EMNODN units have been consistently below the national average, again with wide local variations.

The EMNODN has created a NEC care bundle[8] which was introduced to the 11 neonatal units in September 2022, with use fully embedded by December 2022. Care bundles are a group of evidence-based, simple to implement, interventions related to a disease or care process that, when executed together, result in better outcomes than when implemented individually[9]. The EMNODN NEC care bundle consists of recommendations and parent-facing information to promote own mother's milk feeding and optimise nutritional care of preterm infants. Early start and continuation of feeding with own mother's milk such that

For peer review only - http://bmjopen.bmj.com/site/about/guidelines.xhtml

babies go home breastfeeding is vital to ensure babies continue to receive own mother's milk throughout neonatal care and remain at a reduced risk of NEC.

There is some evidence that quality improvement programmes using care bundles can improve rates of own mother's milk feeding in neonatal units. In the East of England, introduction of a care bundle increased the prevalence of own mother's milk feeding from 50 to 57%[10]. However, no previous studies have explored the effect of complex, multi-component interventions such as a care bundle on rates of NEC and other feeding practices, and there are no published process evaluations of intervention implementation.

Objectives

Our study comprises two parallel, integrated, workstreams with the following objectives:

Workstream 1: Estimate the effects of implementation of the NEC care bundle on rates of own mother's milk feeding and incidence of NEC, comparing infants admitted to the 11 EMNODN neonatal units with admissions to all other units in England and Wales. We will also describe feeding practices before, and after, implementation of the care bundle and examine the infant, maternal and unit-level characteristics associated with own mother's milk feeding and NEC.

Workstream 2: In a multi-centre, mixed methods process evaluation in the 11 EMNODN neonatal units, evaluate how well the care bundle was implemented, understand barriers and facilitators to implementation and understand which components contributed most to effectiveness.

Methods and analysis

Design

Parallel, integrated workstreams:

Workstream 1: Retrospective cohort study using routinely collected clinical data, with interrupted time series analysis.

For peer review only - http://bmjopen.bmj.com/site/about/guidelines.xhtml

Workstream 2: Multi-centre, mixed methods process evaluation based on the RE-AIM[11,12] framework.

Setting

Workstream 1: Neonatal units in England and Wales, comparing infants admitted to the 11 neonatal units in the EMNODN with infants admitted to all other units.

Workstream 2: The 11 neonatal units that comprise the EMNODN.

Time period

01 January 2016 to 30 Sept 2023, with infants grouped by date of birth into three groups according to date of implementation of the care bundle: Pre-implementation period – 01 Jan 2016 to 31 Aug 2022; Implementation period – 01 Sep 2022 to 31 Dec 2022; Evaluation period – 01 Jan 2023 to 30 Sep 2023, followed up for 3 months (until 31 Dec 2023) to cover the full neonatal stay of the final participant.

Data source

Workstream 1: Routinely recorded electronic patient record data from all admissions to NHS neonatal units in England and Wales, held within the National Neonatal Research Database (NNRD). Data are entered by clinical staff at the point of care and extracted, anonymised and combined by the Neonatal Data Analysis Unit (NDAU) at Imperial College, London. The NNRD has NHS Research Ethics Committee approval (10/80803/151) and approval from the Caldicott Guardians of NHS Trusts for research use.

Workstream 2: Quantitative and qualitative data from a variety of sources: questionnaires with parents and HCPs; semi-structured one-to-one interviews with parents and HCPs; NNRD data, supplemented with data directly from paper-based and electronic patient notes, collected using an electronic case report form, for data items not available within the NNRD; data from EMNODN units on cot capacity, staffing levels and facilities.

For peer review only - http://bmjopen.bmj.com/site/about/guidelines.xhtml

Questionnaires will be completed electronically using an online survey platform, with paper copies available should participants request them. Any data from questionnaires completed on paper will be manually digitised, with a sample (10%) checked for verification of data entry. Audio recordings of interviews will be transcribed verbatim and transcripts will be checked against audio recordings to ensure they are accurate and that any identifiable information discussed during interviews is anonymised.

Eligibility criteria

For Workstream 1, we will include infants who: were born at <32 weeks gestational age (GA); survived at least 48 hours from birth; were admitted for neonatal care from 01 January 2016 to 30 September 2023 to a neonatal unit contributing data to the NNRD. In line with previous work[13,14], we will exclude infants with missing data on key characteristics (e.g., sex, gestational age, final discharge destination) as well as infants admitted >12 hours after birth and those missing data for 1 or more episodes of care.

For Workstream 2, we will include parents of infants who are: admitted to one of the 11 EMNODN neonatal units in a 6-month period after implementation of the care bundle; born at <32 weeks GA; survived at least 48 hours from birth. All eligible parents will be invited to respond to the questionnaires. For the interviews, parents will be purposively sampled to include: parents from different neonatal units; parents of extremely preterm infants (born at 22-27 weeks GA) and very preterm infants (born at 28-32 weeks GA); parents whose infants received own mother's milk, mixed feeding, or did not receive any own mothers' milk; parents of infants who had suspected or confirmed NEC; parents representing different maternal (e.g., education) and environmental (including socioeconomic) factors.

We will recruit HCPs working in EMNODN neonatal units. All HCPs will be invited to respond to the questionnaires and some will be purposely sampled to include a range of individuals representing different job roles (unit leads, consultants, junior doctors, nurse practitioners, nurses, health care assistants and allied health professionals including dietitians, speech and language therapists) and hospital sites.

For peer review only - http://bmjopen.bmj.com/site/about/guidelines.xhtml

All questionnaire respondents and interview participants will provide written informed consent for their participation and use of the data provided, including deidentified direct quotations in study outputs.

Intervention

The NEC care bundle, designed by a multi-disciplinary team, consists of recommendations for neonatal units, alongside parent-facing information, to promote own mother's milk feeding and ensure optimum nutritional care of preterm infants. A schematic representation of the care bundle is shown in Figure 1, giving an overview of an infant's journey through neonatal care and opportunities to reduce the risk of NEC.

Recommendations to optimise nutritional care include guidance on: ensuring early and adequate parenteral nutrition given when infants cannot have sufficient milk feeds such as in the first hours after birth, while building milk feeds and when unwell (following established NICE guidelines[15]); use of oropharyngeal colostrum[16] (the practice of giving own mothers' first milk directly into the infant's oropharynx to enhance immunity and protect against NEC); optimal time and speed of increasing milk feeds[17]; and use of other interventions such as human donor milk[18], probiotics[19], and breast milk fortifiers[20].

Primary outcomes

- Receiving any own mother's milk at discharge (exclusively or in combination with infant formula).
- Receiving any own mother's milk on day 14 of life (exclusively or in combination with infant formula).
- Severe NEC requiring surgery or resulting in death[21].

Secondary outcomes

- Exclusive feeding with own mother's milk at discharge.

For peer review only - http://bmjopen.bmj.com/site/about/guidelines.xhtml

- Any NEC, including NEC not requiring surgery and not resulting in death.

Data analysis strategy: Workstream 1

After data cleaning, the number of infants with missing data across key variables will be tabulated and the total number of exclusions and final study population size determined. Descriptive statistics will be presented to summarise the distribution of baseline variables during each of the three study phases, separately for the EMNODN units (intervention group) and all other units in England and Wales (control group). Continuous variables will be reported with means and standard deviations, if shown to be normally distributed using a combined skewness and kurtosis test, or otherwise will be reported using medians and interquartile ranges (IQR). Categorical variables will be reported with frequencies and percentages. P-values for differences between the three time periods will be reported, using the Chi squared test or the Wilcoxon rank-sum test, as appropriate.

We will use interrupted time series (ITS) analysis to assess the impact of implementation of the care bundle on the outcomes listed above, as has been used previously in neonatal research[22]. This is a strong quasi-experimental design which accounts for underlying trends in an outcome. If, for example, rates of NEC were already in decline prior to the implementation of the bundle, the ITS methodology will detect any additional impact of the bundle, above the background decline. It can also account for the impact of any other interventions or changes in population characteristics which might themselves impact outcomes.

We will aggregate outcomes by month of birth (or by two-month period if necessary to improve statistical power, without detriment to interpretation of results) and plot time series graphs to show the number of cases of each outcome per month over the study period in the EMNODN units combined. We will use a quasi-Poisson generalised additive mixed model[23] (GAMM) to estimate an incidence rate ratio (IRR) for changes in the incidence of the

For peer review only - http://bmjopen.bmj.com/site/about/guidelines.xhtml

outcomes in EMNODN units during the evaluation phase, relative to the pre-implementation period. Data for the 4-month implementation phase will be plotted graphically but excluded from statistical modelling as this period is too short to be able to model changes in incidence compared to the pre-implementation period. We will similarly plot and model data for infants admitted to units in the control group, to assess whether there are any changes in the IRR to exclude confounding due to other events or interventions which occurred around the time that the care bundle was introduced.

We will incorporate several covariates into our models as follows:

- The total number of admissions per month will be used as an offset term in each model to account for variations in the number of babies ‘at risk’ of the outcomes.
- The secular trend in each outcome will be captured using a continuous variable coded from 1 to 93 (the total number of months of data across the study period).
- To assess whether there was a step change in the outcomes, we will include a binary variable coded as 0 during the pre-implementation phase and 1 during the evaluation phase.
- To assess whether there was any change in trend (upwards or downwards) in the outcomes, we will include a binary variable coded as 0 during the pre-implementation phase and from 1 to 9 (the number of months in the evaluation phase) during the evaluation phase. We will also assess whether there are any quadratic or cubic trends in the outcomes in the evaluation period, though the relatively short (9 month) length of this evaluation period will limit the ability to detect any non-linear trends. We will use the Akaike Information Criterion (AIC) to compare models with linear, quadratic and cubic trends in the evaluation period, with a smaller AIC indicating better model fit.
- Month of the year will be included as a cyclic cubic spline to capture any non-linear, seasonal patterns in outcomes, such as the impact of activities related to the annual

For peer review only - http://bmjopen.bmj.com/site/about/guidelines.xhtml

World NEC Awareness Day[24] and International Kangaroo Care Awareness Day[25] (both held annually in May).

- To account for any potential impact of the COVID-19 pandemic, we will include a binary variable coded as 1 during periods of national 'lockdown' and 0 otherwise, in line with our previous work examining the impact of pandemic-related visitor restrictions on own mother's milk feeding[26].
- ITS studies are not usually affected by confounders relating to population characteristics as these do not typically change significantly over time[27]. If the characteristics of infants born during each of the three study time periods differ, we will include covariates to capture any differences in potential confounders by month of birth, such as: median GA; percentage of admissions male; median birthweight; percentage of infants with a high-risk NMR-2000 score (a validated score of in-hospital mortality risk)[28]; percentage of infants with a major congenital anomaly; median length of stay.
- Plots of the autocorrelation function (ACF) and partial autocorrelation function (PACF) will be examined to identify plausible values of the autoregressive (AR) and/or moving average (MA) terms to capture autocorrelation between data points. We will use the Akaike Information Criterion (AIC) to compare models with different plausible values of AR and MA terms, with a smaller AIC indicating better model fit.

For peer review only

Multiple testing: IRRs for the primary and secondary outcomes will be reported with 95% confidence intervals and Bonferroni-corrected p-values.

Secondary analyses: In addition to the ITS analysis, we will use multivariable logistic regression to conduct a conventional before-after comparison of the odds of each primary and secondary outcome, adjusted for a range of potential confounders measured at the level of the infant, mother and unit. We will include data from both EMNODN and control units and

For peer review only - http://bmjopen.bmj.com/site/about/guidelines.xhtml

assess whether there is any effect modification by study group. We will use a robust variance estimator to account for the clustering of infants within units and by mother.

Missing data: We will describe the amount of missing data for covariates. For the multivariable logistic regression, we will assume that any missing data occur randomly between the study phases and intervention and control groups and will impute missing values 10-fold using chained equations. Results will be pooled according to Rubin's rule.

Data analysis strategy: Workstream 2

We will use the RE-AIM framework[11,12] to examine implementation of the care bundle, mechanisms of impact and the impact of contextual factors, to map the 'how' and 'why' underpinning the results of the quantitative evaluation undertaken in Workstream 1. We will triangulate data from the NNRD, case report forms, and parent and HCP questionnaires and interviews to address the five evaluative dimensions of RE-AIM:

1) Reach: we will present information on numbers and percentages of infants and parents exposed to different elements of the care bundle and any exceptions or exclusions, considering variation by socio-economic and demographic characteristics. We will measure numbers of HCPs trained to deliver the care bundle and perceptions of the acceptability and usefulness of training.
2) Efficacy: in addition to the overall efficacy assessment in Workstream 1, we will measure the efficacy of key components of the care bundle and investigate their impact on individual level outcomes. For example, we will compare rates of own mother's milk feeding between mothers who received antenatal breastfeeding counselling and mothers who did not.
3) Adoption: with HCPs and unit leads we will explore the contextual factors that vary between units that may impact adoption and fidelity of recommended practices e.g., cost, level of resource, knowledge, training, staff expertise. We will focus on implementation fidelity, using the Framework for Implementation Fidelity (FIF)[29].

For peer review only - http://bmjopen.bmj.com/site/about/guidelines.xhtml

4) Implementation: we will explore factors that may affect implementation of the care bundle using the Consolidated Framework for Implementation Research (CIFR)[30]. This will include factors such as capacity to deliver the care bundle, resources, time, expertise, and leadership buy-in. We will use CIFR to better understand barriers and facilitators to implementation of the bundle.
5) Maintenance: we will assess the extent to which the care bundle is embedded in training, policy and guidance.

Interview data will be analysed using the framework approach[31,32], a hierarchical, matrix-based method to map thematic differences/similarities within and between groups. Data will be coded according to the RE-AIM framework, as well as FIF and CFIR when considering 'Implementation' components, with inductive theme identification within each dimension. Initial reading of transcripts will facilitate familiarisation and generate initial codes. Further reading and immersion will result in substantive themes and sub-themes (the analytical framework). Data will then be indexed according to the identified thematic framework. A sub-sample of transcripts will be double coded to ensure validity. Finally, themes will be discussed and agreed, allowing clarification of the final framework that will be applied across all transcripts. Data will be charted according to each theme to facilitate interpretation, synthesis, and reporting, structured according to the RE-AIM dimensions.

Sample size calculation

Workstream 1: The interrupted time series analysis will have approximately 90% power to detect an absolute increase of 20% in the incidence of receiving own mother's milk at discharge and 80% power to detect a 15% absolute increase (baseline incidence c.55%, c.23 infants per month in the EMNODN from 2017-19). This target is comparable to the incidence of own mother's milk feeding in the best performing neonatal network - in 2021, the South London Neonatal ODN achieved an incidence of 76% (range by unit 71% to 80%) of infants receiving own mother's milk at discharge[33]. In 21 neonatal units in the UK in 2021,

For peer review only

over 75% of infants received own mother's milk at discharge, with the highest performing unit achieving 86% incidence[33].

NEC is a rare occurrence (incidence c.7%, c.3 cases per month in the EMNODN from 2017-19) and there is also considerable monthly variability in incidence. Therefore, statistical power to detect small changes in the incidence of NEC will be more limited; we will be powered to detect approximately a two-thirds reduction in the incidence of NEC. In line with previous studies of less common neonatal outcomes[22], the point estimate will, however, give an indication of the effect of the care bundle, even if the confidence interval around this point estimate crosses the null value.

Workstream 2: We aim to conduct 33 interviews with parents (either individuals or couples, 3 per EMNODN unit) and 33 interviews with HCPs (3 per unit) using a combination of convenience (self-selecting) and purposeful sampling approaches, the latter particularly to approach parents from under-served groups who usually hesitate to participate in research. These numbers are based on principles of maximum variation and theoretical data saturation, which is related to the sampling approach rather than the analysis[34]. Preliminary analysis will inform whether we need to use a second strategy based on the notion of inductive thematic saturation[35] and, in which case, we may need to conduct additional interviews.

Patient, professional and public involvement

The study protocol has been co-produced with the EMNODN parent advisory group (PAG) and the parent-led charity NEC UK[36] to ensure representation from those with lived experiences of NEC and its consequences, both in the neonatal unit and after discharge. The chair of NEC UK is a parent co-applicant and will lead PPI activities. A second PPI representative will also be invited to join the Project Management Group.

For peer review only - http://bmjopen.bmj.com/site/about/guidelines.xhtml

The primary outcome of receiving own mother's milk feeds at discharge was selected by the parents as being the most meaningful outcome for them, and, further to their advice, the emphasis on receiving information and support for breast milk expression in the process evaluation and the plans to explore this in detail in the semi-structured parent interviews were included in the study plan.

With the support of The Centre for Ethnic Health Research[37], a semi-structured focus group discussion has been conducted with women representing different ethnic minority groups, all of whom had experience of having a preterm baby. This discussion focused on co-production of study documentation, particularly the participant information sheets and interview guides. A semi-structured focus group discussion has also been conducted with HCPs working with preterm infants in the East Midlands. This discussion focused on the production of the interview guide for HCPs. Interested parties from both focus groups will be invited to validate qualitative analyses (respondent validation), comment on results and recommendations and review and revise the public-facing dissemination materials. A final parent-led dissemination meeting will be held within the EMNODN.

Ethics and dissemination

The study is sponsored by the University Hospitals of Derby and Burton NHS Foundation Trust and has been registered on the ClinicalTrials.gov database (Ref: NCT05934123). The study has been approved by the South East Scotland Research Ethics Committee 01 and the Health Research Authority and Health and Care Research Wales (IRAS 323099).

We will use the RECORD checklist[38], an extension of the STROBE guidelines[39], to ensure transparency of reporting of our methods and results. On completion of the study, findings will be disseminated via: a detailed report for NIHR as well as the EMNODN and participating neonatal units; public facing materials (infographics, animated video) in English, Polish, Gujarati, and Punjabi (the most frequently-spoken languages in the East Midlands),

For peer review only - http://bmjopen.bmj.com/site/about/guidelines.xhtml

created and shared via parent groups and directly with study participants; open access, peer-reviewed journals and scientific conferences; and dissemination to relevant specialised commissioners including the NHS England Neonatal Critical Care Clinical Reference Group.

Discussion

National recommendations cite a need for quality improvement (QI) efforts to increase own mother's milk feeding to reduce NEC[40]. In 2020, NNAP recommended that if rates of NEC are relatively high, neonatal networks and units should "seek to identify and implement potentially better practices" and to "focus on the early initiation and sustainment of breastmilk feeding in conjunction with parents by reviewing data and processes" and "undertake selected QI activities suited to the local context"[40]. This multi-component evaluation of the care bundle developed for use in the East Midlands will provide evidence to help understand whether the bundle should be rolled out nationally and what changes might be needed to ensure optimum impact and successful implementation within local contexts. Thus, the findings of this study have the potential to improve patient care in the EMNODN and wider, with direct benefits to preterm infants and their families. The parallel effectiveness and process evaluations will establish a framework and toolkit for evaluation of other similar QI initiatives in future.

Patient consent for publication: Not applicable

For peer review only - http://bmjopen.bmj.com/site/about/guidelines.xhtml

Acknowledgements

We acknowledge the East Midlands Neonatal Operational Delivery Network team for their support in setting up the study. We also acknowledge the authors of the EMNODN Care Bundle (Dr Dushyant Batra, Dr Deepa Panjwani, Dr Lara Shipley, Dr Chiara Taylor, Dr Rizma Aishath Moosa, and Ms. Ruth Prigg) for their contribution to the creation of the care bundle.

Electronic patient data recorded at participating neonatal units that collectively form the United Kingdom Neonatal Collaborative are transmitted to the Neonatal Data Analysis Unit to form the National Neonatal Research Database (NNRD). We are grateful to all the families that agreed to the inclusion of their baby's data in the NNRD, the health professionals who recorded data and the Neonatal Data Analysis Unit team.

Author contributions

SO conceptualised and designed the study, contributed to analyses plan, and will lead the delivery and reporting. LS planned the statistical analysis and MB and EO planned the mixed methods process evaluation. JA is the study manager and will perform the qualitative data collection and analysis. HB contributed to study design and will provide input into the interpretation and writing of the results. MS is the PPI Lead and will lead development of public-facing dissemination materials. All authors contributed to the overall study design, development of the protocol and the writing and review of this paper.

Data Statement

Technical appendix, statistical codes, and dataset will be available from the corresponding author.

Funding statement

For peer review only - http://bmjopen.bmj.com/site/about/guidelines.xhtml

This project is funded by the National Institute for Health and Care Research (NIHR) under its Research for Patient Benefit (RfPB) Programme (Grant Reference Number NIHR203590). The views expressed are those of the author(s) and not necessarily those of the NIHR or the Department of Health and Social Care.

Competing interests statement

SO is an Honorary Neonatal Consultant at the University Hospitals of Derby and Burton and is the lead clinician in the team that developed the EMNODN NEC Care Bundle. HB is an Honorary Neonatal Consultant at the Nottingham University Hospitals and was on the advisory panel of the team that developed the EMNODN NEC Care Bundle. MS is the chair of NEC-UK which supports families with experience of NEC and supports breastfeeding mothers. LS, MB, JA and EO have no conflicts of interest to declare.

Word Count: 3,777 words

References

1. Rose, A. T. & Patel, R. M. A critical analysis of risk factors for necrotizing enterocolitis. *Semin. Fetal. Neonatal Med.* **23**, 374–379 (2018).
2. Battersby, C. *et al.* Incidence and enteral feed antecedents of severe neonatal necrotising enterocolitis across neonatal networks in England, 2012-13: a whole-population surveillance study. *Lancet Gastroenterol. Hepatol.* **2**, 43–51 (2017).
3. Adams-Chapman, I. Necrotizing Enterocolitis and Neurodevelopmental Outcome. *Clin. Perinatol.* **45**, 453–466 (2018).

For peer review only - http://bmjopen.bmj.com/site/about/guidelines.xhtml

4. Victora, C. G. *et al.* Breastfeeding in the 21st century: epidemiology, mechanisms, and lifelong effect. *The Lancet* **387**, 475–490 (2016).

5. Mahon, J., Claxton, L. & Wood, H. Modelling the cost-effectiveness of human milk and breastfeeding in preterm infants in the United Kingdom. *Health Econ. Rev.* **6**, 54 (2016).

6. EMNODN. East Midlands Neonatal Operational Delivery Network. https://www.emnodn.nhs.uk.

7. Royal College of Paediatrics and Child Health. National Neonatal Audit Programme. https://www.rcpch.ac.uk/work-we-do/clinical-audits/nnap.

8. East Midlands Neonatal Operational Delivery Network. *Network Guideline: Necrotising enterocolitis care bundle*. https://www.emnodn.nhs.uk/_files/ugd/143840_5050aa59d39b4c66bf2582d7491ba478.pdf (2022).

9. Resar, R., Griffin, F., Haraden, C. & Nolan, T. *Using Care Bundles to Improve Health Care Quality*. (Institute for Healthcare Improvement, 2021).

10. Battersby, C. *et al.* The impact of a regional care bundle on maternal breast milk use in preterm infants: outcomes of the East of England quality improvement programme. *Arch. Dis. Child. Fetal Neonatal Ed.* **99**, F395-401 (2014).

11. Skivington, K. *et al.* A new framework for developing and evaluating complex interventions: update of Medical Research Council guidance. *BMJ* n2061 (2021) doi:10.1136/bmj.n2061.

12. Glasgow, R. E. *et al.* RE-AIM Planning and Evaluation Framework: Adapting to New Science and Practice With a 20-Year Review. *Front. Public Health* **7**, 64 (2019).

13. Sand, L. *et al.* Observational cohort study of changing trends in non-invasive ventilation in very preterm infants and associations with clinical outcomes. *Arch. Dis. Child. Fetal Neonatal Ed.* fetalneonatal-2021-322390 (2021) doi:10.1136/archdischild-2021-322390.

14. Szatkowski, L. *et al.* Observational cohort study of use of caffeine in preterm infants and association between early caffeine use and neonatal outcomes. *Arch. Dis. Child.*

For peer review only - http://bmjopen.bmj.com/site/about/guidelines.xhtml

Fetal Neonatal Ed. fetalneonatal-2022-324919 (2023) doi:10.1136/archdischild-2022-324919.

15. NICE. Neonatal parenteral nutrition NICE guideline [NG154]. (2020).

16. Nasuf, A. W. A., Ojha, S. & Dorling, J. Oropharyngeal colostrum in preventing mortality and morbidity in preterm infants. *Cochrane Database Syst. Rev.* **2019**, (2018).

17. Dorling, J. *et al.* Controlled Trial of Two Incremental Milk-Feeding Rates in Preterm Infants. *N. Engl. J. Med.* **381**, 1434–1443 (2019).

18. Quigley, M., Embleton, N. D. & McGuire, W. Formula versus donor breast milk for feeding preterm or low birth weight infants. *Cochrane Database Syst. Rev.* (2019) doi:10.1002/14651858.CD002971.pub5.

19. Sharif, S., Meader, N., Oddie, S. J., Rojas-Reyes, M. X. & McGuire, W. Probiotics to prevent necrotising enterocolitis in very preterm or very low birth weight infants. *Cochrane Database Syst. Rev.* **2020**, (2020).

20. Brown, J. V. E., Lin, L., Embleton, N. D., Harding, J. E. & McGuire, W. Multi-nutrient fortification of human milk for preterm infants. *Cochrane Database Syst. Rev.* **2020**, (2020).

21. Battersby, C., Santhalingam, T., Costeloe, K. & Modi, N. Incidence of neonatal necrotising enterocolitis in high-income countries: a systematic review. *Arch. Dis. Child. - Fetal Neonatal Ed.* **103**, F182–F189 (2018).

22. Szatkowski, L., McClaughry, R., Clarkson, M. & Sharkey, D. Restricted visiting reduces nosocomial viral respiratory tract infections in high-risk neonates. *Eur. Respir. J.* **53**, 1801874 (2019).

23. Wood, S. N. Fast Stable Restricted Maximum Likelihood and Marginal Likelihood Estimation of Semiparametric Generalized Linear Models. *J. R. Stat. Soc. Ser. B Stat. Methodol.* **73**, 3–36 (2011).

24. NEC UK. NEC Awareness Day. https://www.necuk.org.uk/nec-awareness-day.

25. Kangaroo Mother Care. Kangaroo Care Awareness Day. https://kangaroo.care.

For peer review only - http://bmjopen.bmj.com/site/about/guidelines.xhtml

26. Abdul Hamid, H. B., Szatkowski, L., Budge, H. & Ojha, S. Impact of the coronavirus disease 2019 pandemic on breastfeeding during and at discharge from neonatal care: An observational cohort study. *Pediatr. Investig.* **6**, 189–196 (2022).

27. Bernal, J. L., Cummins, S. & Gasparrini, A. Interrupted time series regression for the evaluation of public health interventions: a tutorial. *Int. J. Epidemiol.* **46**, 348–355 (2017).

28. Medvedev, M. M. *et al.* Development and validation of a simplified score to predict neonatal mortality risk among neonates weighing 2000 g or less (NMR-2000): an analysis using data from the UK and The Gambia. *Lancet Child Adolesc. Health* **4**, 299–311 (2020).

29. Carroll, C. *et al.* A conceptual framework for implementation fidelity. *Implement. Sci. IS* **2**, 40 (2007).

30. Damschroder, L. J., Reardon, C. M., Widerquist, M. A. O. & Lowery, J. The updated Consolidated Framework for Implementation Research based on user feedback. *Implement. Sci.* **17**, 75 (2022).

31. Ritchie, J., Lewis, J. & Elam, G. *Qualitative Research Practice: A Guide for Social Science Students and Researchers*. (Sage, 2003).

32. Gale, N. K., Heath, G., Cameron, E., Rashid, S. & Redwood, S. Using the framework method for the analysis of qualitative data in multi-disciplinary health research. *BMC Med. Res. Methodol.* **13**, 117 (2013).

33. Royal College of Paediatrics and Child Health. *National Neonatal Audit Programme Summary report on 2021 data*. https://www.rcpch.ac.uk/resources/national-neonatal-audit-programme-summary-report-2021-data (2023).

34. Emmel, N. *Sampling and Choosing Cases in Qualitative Research: A Realist Approach*. (SAGE Publications Ltd, 2013). doi:10.4135/9781473913882.

35. Saunders, B. *et al.* Saturation in qualitative research: exploring its conceptualization and operationalization. *Qual. Quant.* **52**, 1893–1907 (2018).

36. NEC UK. https://www.necuk.org.uk.

37. The Centre for Ethnic Health Research. https://ethnichealthresearch.org.uk.

For peer review only - http://bmjopen.bmj.com/site/about/guidelines.xhtml

38. RECORD: REporting of studies Conducted using Observational Routinely-collected Data. https://www.record-statement.org.

39. STROBE: Strengthening the reporting of observational studies in epidemiology. https://www.strobe-statement.org.

40. RCPCH. National Neonatal Audit Programme 2020: Annual Report on 2019 data. (2021).

For peer review only

For peer review only - http://bmjopen.bmj.com/site/about/guidelines.xhtml

Figure 1: Overview of the EMNODN NEC care bundle

For peer review only

For peer review only - http://bmjopen.bmj.com/site/about/guidelines.xhtml

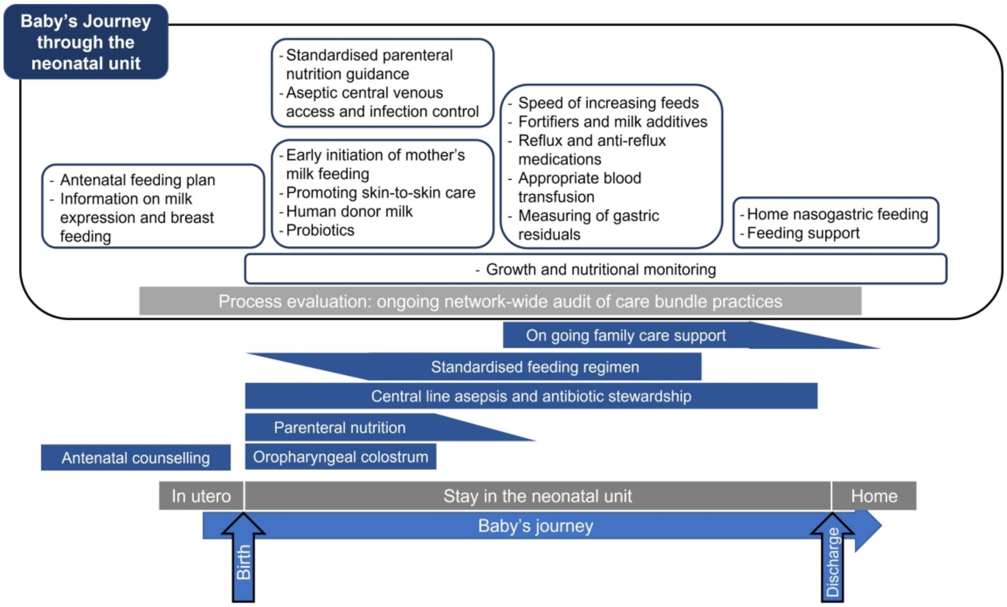

Overview of the EMNODN NEC care bundle

360x217mm (300 x 300 DPI)

BMJ Open

Effects of implementation of a care bundle on rates of necrotising enterocolitis and own mother's milk feeding in the East Midlands: protocol for a mixed methods impact and process evaluation study

Journal:	*BMJ Open*
Manuscript ID	bmjopen-2023-078633.R1
Article Type:	Protocol
Date Submitted by the Author:	01-Feb-2024
Complete List of Authors:	Abramson, Janine; University of Nottingham<br>Szatkowski, Lisa; University of Nottingham, School of Medicine<br>Bains, Manpreet; University of Nottingham, Epidemiology and Public Health<br>Orton, Elizabeth; University of Nottingham<br>Budge, Helen; University of Nottingham; University of Nottingham<br>Spruce, Marie; NEC UK<br>Ojha, Shalini; University of Nottingham; University Hospitals of Derby and Burton NHS Foundation Trust, School of Medicine
**Primary Subject Heading**:	Paediatrics
Secondary Subject Heading:	Evidence based practice, Health services research, Nutrition and metabolism
Keywords:	NUTRITION & DIETETICS, NEONATOLOGY, Nutritional support < GASTROENTEROLOGY

SCHOLARONE™
Manuscripts

Effects of implementation of a care bundle on rates of necrotising enterocolitis and own mother's milk feeding in the East Midlands: protocol for a mixed methods impact and process evaluation study

Janine Abramson[1,2], Lisa Szatkowski[1,2], Manpreet Bains[1], Elizabeth Orton[1], Helen Budge[1,2], Marie Spruce[3], Shalini Ojha[1,2,4]

[1] Lifespan and Population Health, School of Medicine, University of Nottingham, UK

[2] Centre for Perinatal Research, School of Medicine, University of Nottingham, UK

[3] NEC UK, 42 Long Hill Rise, Annesley, Nottingham, NG17 9FG

[4] Neonatal Unit, University Hospitals of Derby and Burton NHS Trust, Derby, UK

Corresponding author: Professor Shalini Ojha, Lifespan and Population Health, School of Medicine, University of Nottingham. Room 4117, Medical School Building, Royal Derby Hospital, Derby DE22 3NE; email: shalini.ojha@nottingham.ac.uk

Orchid ID: https://orcid.org/0000-0001-5668-4227

Key words: infant, preterm; breastfeeding; necrotising enterocolitis

For peer review only

Abstract

300 words

Introduction

Prevention of necrotising enterocolitis (NEC) is vital for improving neonatal outcomes. Feeding own mother's milk helps prevent NEC. Rates of own mother's milk feeding in the East Midlands are lower than the national average and the incidence of NEC is higher. The East Midlands Neonatal Operational Delivery Network (EMNODN) has created a care bundle to improve these in babies born at <32 weeks' gestation, the group at the highest risk of NEC. The bundle was introduced in September 2022 and embedded by December 2022. We will evaluate its effectiveness and conduct a process evaluation to understand barriers and facilitators to implementation.

Methods

In Workstream 1, we will conduct a retrospective cohort study using data from the National Neonatal Research Database (NNRD). We will identify infants receiving any own mother's milk on day 14 and at discharge, and cases of severe NEC. We will aggregate outcomes by birth-month and use interrupted time series analysis to estimate an incidence rate ratio (IRR) for changes after the care bundle was embedded, relative to pre-implementation. We will model data from all other NNRD units and assess whether there are any concurrent changes to exclude confounding due to other events.

In Workstream 2, we will apply the RE-AIM framework, supplemented by the Consolidated Framework for Implementation Research and Framework for Implementation Fidelity, to conduct a mixed methods evaluation in EMNODN units. We will triangulate data from a variety of sources, including questionnaires and semi-structured interviews with parents and healthcare professionals, and data collected from patient records.

For peer review only - http://bmjopen.bmj.com/site/about/guidelines.xhtml

Ethics

South-East Scotland Research Ethics Committee 01.

Dissemination

Disseminated via scientific journals and conferences, to neonatal service commissioners and through public-facing infographics.

Implications

Evidence on the effectiveness of the care bundle will be used to support its maintenance and adoption beyond the East Midlands.

Study registration: ClinicalTrials.gov NCT05934123

For peer review only

For peer review only - http://bmjopen.bmj.com/site/about/guidelines.xhtml

Article Summary

Strengths and limitations of this study

- Use of data from the National Neonatal Research Database (NNRD) allows comparison of all infants admitted to neonatal units in the East Midlands with a control group of all infants admitted to units elsewhere in England and Wales.
- Interrupted time series analysis will allow any changes in own mother's milk feeding and the incidence of NEC related to implementation of the care bundle to be identified over and above any underlying changing trends.
- The parallel mixed methods process evaluation will allow us to unpack the 'how' and 'why' underpinning the results of the interrupted time series analysis.
- Analysis of routine data will allow all eligible infants to participate without risk of under-recruitment from underserved groups. In the process evaluation, we will purposively seek to include parents from underserved groups who traditionally hesitate to participate in research.
- NEC is a rare occurrence and so statistical power to detect small changes in incidence related to implementation of the care bundle may be limited.

For peer review only

Introduction

Necrotising enterocolitis (NEC) is a serious, life-threatening disease in preterm infants[1]. An estimated 12% of infants born weighing <1500g develop NEC and 30% of these die[2]. Survivors have increased risk of adverse neurodevelopmental outcomes, including cerebral palsy and cognitive impairment[3]. Encouraging and supporting own mother's milk feeding prevents NEC and has several additional advantages, including improved long-term neurodevelopment and parent-infant bonding[4]. Health economic analysis estimates that if all premature infants were to be fed own mother's milk, the total lifetime cost saving to the NHS would be £46.7 million (£30.1 million in the first year of life) and there would be 238 fewer deaths per year with a gain of £153.4 million in lifetime productivity[5].

Neonatal critical care services in England are commissioned by NHS England and managed within 11 Neonatal Operational Delivery Networks (ODNs), to ensure that babies and their families receive high quality, equitable and accessible care. The East Midlands Neonatal Operational Delivery Network (EMNODN[6]) comprises 11 neonatal units across 7 NHS Trusts, covering an area of 15,627km^2, a total population of approximately 4.9 million[7] and approximately 47,000 live births per year[8]. In 2022, EMNODN units provided 50,443 days of care in total to infants born preterm, or unwell at term, who needed specialist neonatal care; 476 infants were cared for who were born before 32 weeks' gestational age and survived at least 48 hours[9].

The rate of NEC in the EMNODN has remained above the national average since 2017, when this measure was added to the National Neonatal Audit Programme, NNAP[10]. Additionally, there are wide variations between the 11 units that comprise the EMNODN. Rates of any breastmilk feeding at discharge from EMNODN units have been consistently below the national average, again with wide local variations.

For peer review only - http://bmjopen.bmj.com/site/about/guidelines.xhtml

The EMNODN has created a NEC care bundle[11] which was introduced to the 11 neonatal units in September 2022, with use fully embedded by December 2022. Care bundles are a group of evidence-based, simple to implement, interventions related to a disease or care process that, when executed together, result in better outcomes than when implemented individually[12]. The EMNODN NEC care bundle consists of recommendations and parent-facing information to promote own mother's milk feeding and optimise nutritional care of preterm infants. Early start and continuation of feeding with own mother's milk such that babies go home breastfeeding is vital to ensure babies continue to receive own mother's milk throughout neonatal care and remain at a reduced risk of NEC.

There is some evidence that quality improvement programmes using care bundles can improve rates of own mother's milk feeding in neonatal units. In the East of England, introduction of a care bundle increased the prevalence of own mother's milk feeding from 50 to 57%[13]. However, no previous studies have explored the effect of complex, multi-component interventions such as a care bundle on rates of NEC and other feeding practices, and there are no published process evaluations of intervention implementation.

Objectives

Our study comprises two parallel, integrated, workstreams with the following objectives:

Workstream 1: Estimate the effects of implementation of the NEC care bundle on rates of own mother's milk feeding and incidence of NEC, comparing infants admitted to the 11 EMNODN neonatal units with admissions to all other units in England and Wales. We will also describe feeding practices before, and after, implementation of the care bundle and examine the infant, maternal and unit-level characteristics associated with own mother's milk feeding and NEC.

Workstream 2: In a multi-centre, mixed methods process evaluation in the 11 EMNODN neonatal units, evaluate how well the care bundle was implemented, understand barriers

For peer review only - http://bmjopen.bmj.com/site/about/guidelines.xhtml

and facilitators to implementation and understand which components contributed most to effectiveness.

Methods and analysis

Design

Parallel, integrated workstreams:

Workstream 1: Retrospective cohort study using routinely collected clinical data, with interrupted time series analysis.

Workstream 2: Multi-centre, mixed methods process evaluation based on the RE-AIM Framework[14,15], Consolidated Framework for Implementation Research (CFIR)[16] and Framework for Implementation Fidelity (FIF)[17].

Setting

Workstream 1: Neonatal units in England and Wales, comparing infants admitted to the 11 neonatal units in the EMNODN with infants admitted to all other units.

Workstream 2: The 11 neonatal units that comprise the EMNODN.

Time period

01 January 2016 to 30 Sept 2023, with infants grouped by date of birth into three groups according to date of implementation of the care bundle: Pre-implementation period – 01 Jan 2016 to 31 Aug 2022; Implementation period – 01 Sep 2022 to 31 Dec 2022; Evaluation period – 01 Jan 2023 to 30 Sep 2023, followed up for 3 months (until 31 Dec 2023) to cover the full neonatal stay of the final participant.

Data source

Workstream 1: Routinely recorded electronic patient record data from all admissions to NHS neonatal units in England and Wales, held within the National Neonatal Research Database (NNRD). Data are entered by clinical staff at the point of care and extracted, anonymised

For peer review only - http://bmjopen.bmj.com/site/about/guidelines.xhtml

and combined by the Neonatal Data Analysis Unit (NDAU) at Imperial College, London. The NNRD has NHS Research Ethics Committee approval (10/80803/151) and approval from the Caldicott Guardians of NHS Trusts for research use.

Workstream 2: Quantitative and qualitative data from a variety of sources: questionnaires with parents and HCPs; semi-structured one-to-one interviews with parents and HCPs; NNRD data, supplemented with data directly from paper-based and electronic patient notes, collected using an electronic case report form, for data items not available within the NNRD; data from EMNODN units on cot capacity, staffing levels and facilities.

Questionnaires will be completed electronically using an online survey platform, with paper copies available should participants request them. Any data from questionnaires completed on paper will be manually digitised, with a sample (10%) checked for verification of data entry. Audio recordings of interviews will be transcribed verbatim and transcripts will be checked against audio recordings to ensure they are accurate and that any identifiable information discussed during interviews is anonymised.

Eligibility criteria

For Workstream 1, we will include infants who: were born at <32 weeks gestational age (GA); survived at least 48 hours from birth; were admitted for neonatal care from 01 January 2016 to 30 September 2023 to a neonatal unit contributing data to the NNRD. In line with previous work[18,19], we will exclude infants with missing data on key characteristics (e.g., sex, gestational age, final discharge destination) as well as infants admitted >12 hours after birth and those missing data for 1 or more episodes of care.

For Workstream 2, we will include parents of infants who are: admitted to one of the 11 EMNODN neonatal units in a 6-month period after implementation of the care bundle; born at <32 weeks GA; survived at least 48 hours from birth. All eligible parents will be invited to respond to the questionnaires. For the interviews, parents will be purposively sampled to

For peer review only - http://bmjopen.bmj.com/site/about/guidelines.xhtml

include: parents from different neonatal units; parents of extremely preterm infants (born at 22-27 weeks GA) and very preterm infants (born at 28-32 weeks GA); parents whose infants received own mother's milk, mixed feeding, or did not receive any own mothers' milk; parents of infants who had suspected or confirmed NEC; parents representing different maternal (e.g., education) and environmental (including socioeconomic) factors.

We will recruit HCPs working in EMNODN neonatal units. All HCPs will be invited to respond to the questionnaires and some will be purposely sampled to include a range of individuals representing different job roles (unit leads, consultants, junior doctors, nurse practitioners, nurses, health care assistants and allied health professionals including dietitians, speech and language therapists) and hospital sites.

All questionnaire respondents and interview participants will provide written informed consent for their participation and use of the data provided, including deidentified direct quotations in study outputs.

Intervention

The NEC care bundle, designed by a multi-disciplinary team, consists of recommendations for neonatal units, alongside parent-facing information, to promote own mother's milk feeding and ensure optimum nutritional care of preterm infants. A schematic representation of the care bundle is shown in Figure 1, giving an overview of an infant's journey through neonatal care and opportunities to reduce the risk of NEC.

Recommendations to optimise nutritional care include guidance on: ensuring early and adequate parenteral nutrition given when infants cannot have sufficient milk feeds such as in the first hours after birth, while building milk feeds and when unwell (following established NICE guidelines[20]); use of oropharyngeal colostrum[21] (the practice of giving own mothers' first milk directly into the infant's oropharynx to enhance immunity and protect against NEC); optimal time and speed of increasing milk feeds[22]; and use of other interventions such as human donor milk[23], probiotics[24], and breast milk fortifiers[25].

For peer review only - http://bmjopen.bmj.com/site/about/guidelines.xhtml

Primary outcomes

- Receiving any own mother's milk at discharge (exclusively or in combination with infant formula).
- Receiving any own mother's milk on day 14 of life (exclusively or in combination with infant formula).
- Severe NEC requiring surgery or resulting in death[26].

Secondary outcomes

- Exclusive feeding with own mother's milk at discharge.
- Any NEC, including NEC not requiring surgery and not resulting in death.

Data analysis strategy: Workstream 1

After data cleaning, the number of infants with missing data across key variables will be tabulated and the total number of exclusions and final study population size determined. Descriptive statistics will be presented to summarise the distribution of baseline variables during each of the three study phases, separately for the EMNODN units (intervention group) and all other units in England and Wales (control group). Continuous variables will be reported with means and standard deviations, if shown to be normally distributed using a combined skewness and kurtosis test, or otherwise will be reported using medians and interquartile ranges (IQR). Categorical variables will be reported with frequencies and percentages. P-values for differences between the three time periods will be reported, using the Chi squared test or the Wilcoxon rank-sum test, as appropriate.

We will use interrupted time series (ITS) analysis to assess the impact of implementation of the care bundle on the outcomes listed above, as has been used previously in neonatal research[27]. This is a strong quasi-experimental design which accounts for underlying trends in an outcome. If, for example, rates of NEC were already in decline prior to the

For peer review only - http://bmjopen.bmj.com/site/about/guidelines.xhtml

implementation of the bundle, the ITS methodology will detect any additional impact of the bundle, above the background decline. It can also account for the impact of any other interventions or changes in population characteristics which might themselves impact outcomes.

We will aggregate outcomes by month of birth (or by two-month period if necessary to improve statistical power, without detriment to interpretation of results) and plot time series graphs to show the number of cases of each outcome per month over the study period in the EMNODN units combined. We will use a quasi-Poisson generalised additive mixed model[28] (GAMM) to estimate an incidence rate ratio (IRR) for changes in the incidence of the outcomes in EMNODN units during the evaluation phase, relative to the pre-implementation period. Data for the 4-month implementation phase will be plotted graphically but excluded from statistical modelling as this period is too short to be able to model changes in incidence compared to the pre-implementation period. We will similarly plot and model data for infants admitted to units in the control group, to assess whether there are any changes in the IRR to exclude confounding due to other events or interventions which occurred around the time that the care bundle was introduced.

We will incorporate several covariates into our models as follows:

- The total number of admissions per month will be used as an offset term in each model to account for variations in the number of babies ‘at risk’ of the outcomes.
- The secular trend in each outcome will be captured using a continuous variable coded from 1 to 93 (the total number of months of data across the study period).
- To assess whether there was a step change in the outcomes, we will include a binary variable coded as 0 during the pre-implementation phase and 1 during the evaluation phase.
- To assess whether there was any change in trend (upwards or downwards) in the outcomes, we will include a binary variable coded as 0 during the pre-implementation

For peer review only - http://bmjopen.bmj.com/site/about/guidelines.xhtml

phase and from 1 to 9 (the number of months in the evaluation phase) during the evaluation phase. We will also assess whether there are any quadratic or cubic trends in the outcomes in the evaluation period, though the relatively short (9 month) length of this evaluation period will limit the ability to detect any non-linear trends. We will use the Akaike Information Criterion (AIC) to compare models with linear, quadratic and cubic trends in the evaluation period, with a smaller AIC indicating better model fit.

- Month of the year will be included as a cyclic cubic spline to capture any non-linear, seasonal patterns in outcomes, such as the impact of activities related to the annual World NEC Awareness Day[29] and International Kangaroo Care Awareness Day[30] (both held annually in May), or any variation in NEC incidence related to seasonality of circulating pathogens[31].
- To account for any potential impact of the COVID-19 pandemic, we will include a binary variable coded as 1 during periods of national 'lockdown' and 0 otherwise, in line with our previous work examining the impact of pandemic-related visitor restrictions on own mother's milk feeding[32].
- ITS studies are not usually affected by confounders relating to population characteristics as these do not typically change significantly over time[33]. If the characteristics of infants born during each of the three study time periods differ, we will include covariates to capture any differences in potential confounders by month of birth, such as: median GA; percentage of admissions male; median birthweight; percentage of infants with a high-risk NMR-2000 score (a validated score of in-hospital mortality risk)[34]; percentage of infants with a major congenital anomaly; median length of stay.
- Plots of the autocorrelation function (ACF) and partial autocorrelation function (PACF) will be examined to identify plausible values of the autoregressive (AR) and/or moving average (MA) terms to capture autocorrelation between data points.

For peer review only - http://bmjopen.bmj.com/site/about/guidelines.xhtml

For peer review only

We will use the Akaike Information Criterion (AIC) to compare models with different plausible values of AR and MA terms, with a smaller AIC indicating better model fit.

Multiple testing: IRRs for the primary and secondary outcomes will be reported with 95% confidence intervals and Bonferroni-corrected p-values.

Secondary analyses: In addition to the ITS analysis, we will use multivariable logistic regression to conduct a conventional before-after comparison of the odds of each primary and secondary outcome, adjusted for a range of potential confounders measured at the level of the infant, mother and unit. We will include data from both EMNODN and control units and assess whether there is any effect modification by study group. We will use a robust variance estimator to account for the clustering of infants within units and by mother.

Missing data: We will describe the amount of missing data for covariates. For the multivariable logistic regression, we will assume that any missing data occur randomly between the study phases and intervention and control groups and will impute missing values 10-fold using chained equations. Results will be pooled according to Rubin's rule.

Data analysis strategy: Workstream 2

We will use the RE-AIM framework[14,15], supplemented by the Consolidated Framework for Implementation Research (CFIR)[16] and the Framework for Intervention Fidelity (FIF)[17], to examine implementation of the care bundle, mechanisms of impact and the impact of contextual factors, to map the 'how' and 'why' underpinning the results of the quantitative evaluation undertaken in Workstream 1. These frameworks bring together metrics for evaluating implementation success, and by assessing reach and effectiveness the frameworks can be used together to identify ways to produce sustained changes (RE-AIM and FIF), which will highlight modifiable factors that explain and enhance implementation outcomes (CFIR). We will triangulate data from the NNRD, case report

For peer review only - http://bmjopen.bmj.com/site/about/guidelines.xhtml

For peer review only

forms, and parent and HCP questionnaires and interviews to address the five evaluative dimensions of RE-AIM and map the CFIR constructs and FIF checklist within these domains (as appropriate e.g., particularly around Adoption, Implementation and Maintenance), to understand factors impacting implementation:

1) Reach: we will present information on numbers and percentages of infants and parents exposed to different elements of the care bundle and any exceptions or exclusions, considering variation by socio-economic and demographic characteristics. We will measure numbers of HCPs trained to deliver the care bundle and perceptions of the acceptability and usefulness of training.
2) Efficacy: in addition to the overall efficacy assessment in Workstream 1, we will measure the efficacy of key components of the care bundle and investigate their impact on individual level outcomes. For example, we will compare rates of own mother's milk feeding between mothers who received antenatal breastfeeding counselling and mothers who did not.
3) Adoption: with HCPs and unit leads we will explore the contextual factors that vary between units that may impact adoption and fidelity of recommended practices, using the FIF checklist and CFIR to guide thematic coding. Moderators of adoption and fidelity may include staff training and existing expertise, level of available resource locally.
4) Implementation: we will explore factors that may affect implementation of the care bundle, using CFIR as a coding framework. This will include factors related to the care bundle (e.g., its complexity and evidence-base), factors related to setting (e.g., staff capacity to deliver the care bundle, availability of resources, time and expertise, and leadership buy-in), attributes of roles within the teams and their motivations to implement the care bundle, and if and how the care bundle is adapted in each setting. This will help us better understand barriers and facilitators to implementation of the bundle.
5) Maintenance: we will assess the extent to which the care bundle is embedded in training, policy and guidance.

For peer review only - http://bmjopen.bmj.com/site/about/guidelines.xhtml

Interview data will be analysed using the framework approach[35,36], a hierarchical, matrix-based method to map thematic differences/similarities within and between groups. First, a priori codes relating to the RE-AIM domains (Adoption, Implementation and Maintenance) will be applied to the data. Transcripts will then be coded again to apply the CFIR domains, constructs or subconstructs and FIF checklist (identified to influence RE-AIM domains), inductively. As an initial step to check validity of coding, two researchers will compare coding of several transcripts to reach consensus on interpretations. The remaining data will then be coded according to the relevant RE-AIM, CFIR and FIF code domains and construct codes, and the generated themes, with matrices of results and quotes presented and discussed among the full research team, to agree on data interpretation and the final themes.

Sample size calculation

Workstream 1: The interrupted time series analysis will have approximately 90% power to detect an absolute increase of 20% in the prevalence of receiving own mother's milk at discharge and 80% power to detect a 15% absolute increase (baseline prevalence c.55%, c.23 infants per month in the EMNODN from 2017-19; national average c.60%). This target is comparable to the prevalence of own mother's milk feeding in the best performing neonatal network - in 2021, the South London Neonatal ODN achieved a prevalence of 76% (range by unit 71% to 80%) of infants receiving own mother's milk at discharge[37]. In 21 neonatal units in the UK in 2021, over 75% of infants received own mother's milk at discharge, with the highest performing unit achieving 86% prevalence[37].

NEC is a rare occurrence (incidence c.7%, c.3 cases per month in the EMNODN from 2017-19; national average c.5%) and there is also considerable monthly variability in incidence. Therefore, statistical power to detect small changes in the incidence of NEC will be more limited; we will be powered to detect approximately a two-thirds reduction in the incidence of NEC. In line with previous studies of less common neonatal outcomes[27], the point estimate

For peer review only - http://bmjopen.bmj.com/site/about/guidelines.xhtml

will, however, give an indication of the effect of the care bundle, even if the confidence interval around this point estimate crosses the null value.

Workstream 2: We aim to conduct 33 interviews with parents (either individuals or couples, 3 per EMNODN unit) and 33 interviews with HCPs (3 per unit) using a combination of convenience (self-selecting) and purposeful sampling approaches, the latter particularly to approach parents from under-served groups who usually hesitate to participate in research. These numbers are based on principles of maximum variation and theoretical data saturation, which is related to the sampling approach rather than the analysis[38]. Preliminary analysis will inform whether we need to use a second strategy based on the notion of inductive thematic saturation[39] and, in which case, we may need to conduct additional interviews.

Patient and Public Involvement

The study protocol has been co-produced with the EMNODN parent advisory group (PAG) and the parent-led charity NEC UK[40] to ensure representation from those with lived experiences of NEC and its consequences, both in the neonatal unit and after discharge. The chair of NEC UK is a parent co-applicant and will lead PPI activities. A second PPI representative will also be invited to join the Project Management Group.

The primary outcome of receiving own mother's milk feeds at discharge was selected by the parents as being the most meaningful outcome for them, and, further to their advice, the emphasis on receiving information and support for breast milk expression in the process evaluation and the plans to explore this in detail in the semi-structured parent interviews were included in the study plan.

For peer review only - http://bmjopen.bmj.com/site/about/guidelines.xhtml

With the support of The Centre for Ethnic Health Research[41], a semi-structured focus group discussion has been conducted with women representing different ethnic minority groups, all of whom had experience of having a preterm baby. This discussion focused on co-production of study documentation, particularly the participant information sheets and interview guides. A semi-structured focus group discussion has also been conducted with HCPs working with preterm infants in the East Midlands. This discussion focused on the production of the interview guide for HCPs. Interested parties from both focus groups will be invited to validate qualitative analyses (respondent validation), comment on results and recommendations and review and revise the public-facing dissemination materials. A final parent-led dissemination meeting will be held within the EMNODN.

Ethics and dissemination

The study is sponsored by the University Hospitals of Derby and Burton NHS Foundation Trust and has been registered on the ClinicalTrials.gov database (Ref: NCT05934123). The study has been approved by the South East Scotland Research Ethics Committee 01 and the Health Research Authority and Health and Care Research Wales (IRAS 323099).

We will use the RECORD checklist[42], an extension of the STROBE guidelines[43], to ensure transparency of reporting of our methods and results. On completion of the study, findings will be disseminated via: a detailed report for NIHR as well as the EMNODN and participating neonatal units; public facing materials (infographics, animated video) in English, Polish, Gujarati, and Punjabi (the most frequently-spoken languages in the East Midlands), created and shared via parent groups and directly with study participants; open access, peer-reviewed journals and scientific conferences; and dissemination to relevant specialised commissioners including the NHS England Neonatal Critical Care Clinical Reference Group.

For peer review only

For peer review only - http://bmjopen.bmj.com/site/about/guidelines.xhtml

Discussion

National recommendations cite a need for quality improvement (QI) efforts to increase own mother's milk feeding to reduce NEC[44]. In 2020, NNAP recommended that if rates of NEC are relatively high, neonatal networks and units should "seek to identify and implement potentially better practices" and to "focus on the early initiation and sustainment of breastmilk feeding in conjunction with parents by reviewing data and processes" and "undertake selected QI activities suited to the local context"[44]. This multi-component evaluation of the care bundle developed for use in the East Midlands will provide evidence to help understand whether the bundle should be rolled out nationally and what changes might be needed to ensure optimum impact and successful implementation within local contexts. Thus, the findings of this study have the potential to improve patient care in the EMNODN and wider, with direct benefits to preterm infants and their families. The parallel effectiveness and process evaluations will establish a framework and toolkit for evaluation of other similar QI initiatives in future.

Patient consent for publication: Not applicable

Acknowledgements

We acknowledge the East Midlands Neonatal Operational Delivery Network team for their support in setting up the study. We also acknowledge the authors of the EMNODN Care Bundle (Dr Dushyant Batra, Dr Deepa Panjwani, Dr Lara Shipley, Dr Chiara Taylor, Dr Rizma Aishath Moosa, and Ms. Ruth Prigg) for their contribution to the creation of the care bundle.

Electronic patient data recorded at participating neonatal units that collectively form the United Kingdom Neonatal Collaborative are transmitted to the Neonatal Data Analysis Unit to form the National Neonatal Research Database (NNRD). We are grateful to all the

For peer review only - http://bmjopen.bmj.com/site/about/guidelines.xhtml

families that agreed to the inclusion of their baby's data in the NNRD, the health professionals who recorded data and the Neonatal Data Analysis Unit team.

Author contributions

SO conceptualised and designed the study, contributed to analyses plan, and will lead the delivery and reporting. LS planned the statistical analysis and MB and EO planned the mixed methods process evaluation. JA is the study manager and will perform the qualitative data collection and analysis. HB contributed to study design and will provide input into the interpretation and writing of the results. MS is the PPI Lead and will lead development of public-facing dissemination materials. All authors contributed to the overall study design, development of the protocol and the writing and review of this paper.

Data Statement

Technical appendix, statistical codes, and dataset will be available from the corresponding author.

Funding statement

This project is funded by the National Institute for Health and Care Research (NIHR) under its Research for Patient Benefit (RfPB) Programme (Grant Reference Number NIHR203590). The views expressed are those of the author(s) and not necessarily those of the NIHR or the Department of Health and Social Care.

Competing interests statement

For peer review only

SO is an Honorary Neonatal Consultant at the University Hospitals of Derby and Burton and is the lead clinician in the team that developed the EMNODN NEC Care Bundle. HB is an Honorary Neonatal Consultant at the Nottingham University Hospitals and was on the advisory panel of the team that developed the EMNODN NEC Care Bundle. MS is the chair of NEC-UK which supports families with experience of NEC and supports breastfeeding mothers. LS, MB, JA and EO have no conflicts of interest to declare.

For peer review only

Word Count: 3,777 words

References

1. Rose AT, Patel RM. A critical analysis of risk factors for necrotizing enterocolitis. Semin Fetal Neonatal Med. 2018;23: 374–379. doi:10.1016/j.siny.2018.07.005

2. Battersby C, Longford N, Mandalia S, Costeloe K, Modi N, UK Neonatal Collaborative Necrotising Enterocolitis (UKNC-NEC) study group. Incidence and enteral feed antecedents of severe neonatal necrotising enterocolitis across neonatal networks in England, 2012-13: a whole-population surveillance study. Lancet Gastroenterol Hepatol. 2017;2: 43–51. doi:10.1016/S2468-1253(16)30117-0

3. Adams-Chapman I. Necrotizing Enterocolitis and Neurodevelopmental Outcome. Clin Perinatol. 2018;45: 453–466. doi:10.1016/j.clp.2018.05.014

4. Victora CG, Bahl R, Barros AJD, França GVA, Horton S, Krasevec J, et al. Breastfeeding in the 21st century: epidemiology, mechanisms, and lifelong effect. The Lancet. 2016;387: 475–490. doi:10.1016/S0140-6736(15)01024-7

5. Mahon J, Claxton L, Wood H. Modelling the cost-effectiveness of human milk and breastfeeding in preterm infants in the United Kingdom. Health Econ Rev. 2016;6: 54. doi:10.1186/s13561-016-0136-0

6. EMNODN. East Midlands Neonatal Operational Delivery Network. [cited 20 Apr 2023]. Available: https://www.emnodn.nhs.uk

7. Office for National Statistics. 2021 Census Profile for areas in England and Wales: East Midlands Region. [cited 10 Jan 2024]. Available: https://www.nomisweb.co.uk/sources/census_2021/report?compare=E12000004

8. Office for National Statistics. Births in England and Wales: 2022. [cited 11 Jan 2024]. Available:

For peer review only - http://bmjopen.bmj.com/site/about/guidelines.xhtml

https://www.ons.gov.uk/peoplepopulationandcommunity/birthsdeathsandmarriages/livebirths/bulletins/birthsummarytablesenglandandwales/2022

9. Royal College of Paediatrics and Child Health. Neonatal unit and neonatal network annual summary reports. [cited 10 Dec 2023]. Available: https://nnap.rcpch.ac.uk/annual-reports.aspx

10. Royal College of Paediatrics and Child Health. National Neonatal Audit Programme. [cited 20 Apr 2023]. Available: https://www.rcpch.ac.uk/work-we-do/clinical-audits/nnap

11. East Midlands Neonatal Operational Delivery Network. Network Guideline: Necrotising enterocolitis care bundle. 2022. Available: https://www.emnodn.nhs.uk/_files/ugd/143840_5050aa59d39b4c66bf2582d7491ba478.pdf

12. Resar R, Griffin F, Haraden C, Nolan T. Using Care Bundles to Improve Health Care Quality. Cambridge, Massachusetts: Institute for Healthcare Improvement; 2021. Available: http://www.ihi.org/resources/Pages/IHIWhitePapers/UsingCareBundles.aspx

13. Battersby C, Santhakumaran S, Upton M, Radbone L, Birch J, Modi N, et al. The impact of a regional care bundle on maternal breast milk use in preterm infants: outcomes of the East of England quality improvement programme. Arch Dis Child Fetal Neonatal Ed. 2014;99: F395-401. doi:10.1136/archdischild-2013-305475

14. Skivington K, Matthews L, Simpson SA, Craig P, Baird J, Blazeby JM, et al. A new framework for developing and evaluating complex interventions: update of Medical Research Council guidance. BMJ. 2021; n2061. doi:10.1136/bmj.n2061

15. Glasgow RE, Harden SM, Gaglio B, Rabin B, Smith ML, Porter GC, et al. RE-AIM Planning and Evaluation Framework: Adapting to New Science and Practice With a 20-Year Review. Front Public Health. 2019;7: 64. doi:10.3389/fpubh.2019.00064

16. Damschroder LJ, Reardon CM, Widerquist MAO, Lowery J. The updated Consolidated Framework for Implementation Research based on user feedback. Implement Sci. 2022;17: 75. doi:10.1186/s13012-022-01245-0

17. Carroll C, Patterson M, Wood S, Booth A, Rick J, Balain S. A conceptual framework for implementation fidelity. Implement Sci IS. 2007;2: 40. doi:10.1186/1748-5908-2-40

18. Sand L, Szatkowski L, Kwok TC, Sharkey D, Todd DA, Budge H, et al. Observational cohort study of changing trends in non-invasive ventilation in very preterm infants and associations with clinical outcomes. Arch Dis Child Fetal Neonatal Ed. 2021; fetalneonatal-2021-322390. doi:10.1136/archdischild-2021-322390

19. Szatkowski L, Fateh S, Abramson J, Kwok TC, Sharkey D, Budge H, et al. Observational cohort study of use of caffeine in preterm infants and association between early caffeine use and neonatal outcomes. Arch Dis Child Fetal Neonatal Ed. 2023; fetalneonatal-2022-324919. doi:10.1136/archdischild-2022-324919

20. NICE. Neonatal parenteral nutrition NICE guideline [NG154]. 2020. Available: https://www.nice.org.uk/guidance/ng154

21. Nasuf AWA, Ojha S, Dorling J. Oropharyngeal colostrum in preventing mortality and morbidity in preterm infants. Cochrane Neonatal Group, editor. Cochrane Database Syst Rev. 2018;2019. doi:10.1002/14651858.CD011921.pub2

For peer review only

22. Dorling J, Abbott J, Berrington J, Bosiak B, Bowler U, Boyle E, et al. Controlled Trial of Two Incremental Milk-Feeding Rates in Preterm Infants. N Engl J Med. 2019;381: 1434–1443. doi:10.1056/NEJMoa1816654

23. Quigley M, Embleton ND, McGuire W. Formula versus donor breast milk for feeding preterm or low birth weight infants. Cochrane Neonatal Group, editor. Cochrane Database Syst Rev. 2019 [cited 17 Nov 2021]. doi:10.1002/14651858.CD002971.pub5

24. Sharif S, Meader N, Oddie SJ, Rojas-Reyes MX, McGuire W. Probiotics to prevent necrotising enterocolitis in very preterm or very low birth weight infants. Cochrane Neonatal Group, editor. Cochrane Database Syst Rev. 2020;2020. doi:10.1002/14651858.CD005496.pub5

25. Brown JVE, Lin L, Embleton ND, Harding JE, McGuire W. Multi-nutrient fortification of human milk for preterm infants. Cochrane Neonatal Group, editor. Cochrane Database Syst Rev. 2020;2020. doi:10.1002/14651858.CD000343.pub4

26. Battersby C, Santhalingam T, Costeloe K, Modi N. Incidence of neonatal necrotising enterocolitis in high-income countries: a systematic review. Arch Dis Child - Fetal Neonatal Ed. 2018;103: F182–F189. doi:10.1136/archdischild-2017-313880

27. Szatkowski L, McClaughry R, Clarkson M, Sharkey D. Restricted visiting reduces nosocomial viral respiratory tract infections in high-risk neonates. Eur Respir J. 2019;53: 1801874. doi:10.1183/13993003.01874-2018

28. Wood SN. Fast Stable Restricted Maximum Likelihood and Marginal Likelihood Estimation of Semiparametric Generalized Linear Models. J R Stat Soc Ser B Stat Methodol. 2011;73: 3–36. doi:10.1111/j.1467-9868.2010.00749.x

29. NEC UK. NEC Awareness Day. [cited 12 May 2023]. Available: https://www.necuk.org.uk/nec-awareness-day

30. Kangaroo Mother Care. Kangaroo Care Awareness Day. [cited 12 May 2023]. Available: https://kangaroo.care

31. Snyder CL, Hall M, Sharma V, St. Peter SD. Seasonal variation in the incidence of necrotizing enterocolitis. Pediatr Surg Int. 2010;26: 895–898. doi:10.1007/s00383-010-2675-5

32. Abdul Hamid HB, Szatkowski L, Budge H, Ojha S. Impact of the coronavirus disease 2019 pandemic on breastfeeding during and at discharge from neonatal care: An observational cohort study. Pediatr Investig. 2022;6: 189–196. doi:10.1002/ped4.12337

33. Bernal JL, Cummins S, Gasparrini A. Interrupted time series regression for the evaluation of public health interventions: a tutorial. Int J Epidemiol. 2017;46: 348–355. doi:10.1093/ije/dyw098

34. Medvedev MM, Brotherton H, Gai A, Tann C, Gale C, Waiswa P, et al. Development and validation of a simplified score to predict neonatal mortality risk among neonates weighing 2000 g or less (NMR-2000): an analysis using data from the UK and The Gambia. Lancet Child Adolesc Health. 2020;4: 299–311. doi:10.1016/S2352-4642(20)30021-3

35. Ritchie J, Lewis J, Elam G. Qualitative Research Practice: A Guide for Social Science Students and Researchers. London: Sage; 2003.

For peer review only - http://bmjopen.bmj.com/site/about/guidelines.xhtml

36. Gale NK, Heath G, Cameron E, Rashid S, Redwood S. Using the framework method for the analysis of qualitative data in multi-disciplinary health research. BMC Med Res Methodol. 2013;13: 117. doi:10.1186/1471-2288-13-117

37. Royal College of Paediatrics and Child Health. National Neonatal Audit Programme Summary report on 2021 data. 2023. Available: https://www.rcpch.ac.uk/resources/national-neonatal-audit-programme-summary-report-2021-data

38. Emmel N. Sampling and Choosing Cases in Qualitative Research: A Realist Approach. 1 Oliver's Yard, 55 City Road, London EC1Y 1SP United Kingdom: SAGE Publications Ltd; 2013. doi:10.4135/9781473913882

39. Saunders B, Sim J, Kingstone T, Baker S, Waterfield J, Bartlam B, et al. Saturation in qualitative research: exploring its conceptualization and operationalization. Qual Quant. 2018;52: 1893–1907. doi:10.1007/s11135-017-0574-8

40. NEC UK. [cited 30 Apr 2023]. Available: https://www.necuk.org.uk

41. The Centre for Ethnic Health Research. [cited 30 Apr 2023]. Available: https://ethnichealthresearch.org.uk

42. RECORD: REporting of studies Conducted using Observational Routinely-collected Data. [cited 1 Jun 2023]. Available: https://www.record-statement.org

43. STROBE: Strengthening the reporting of observational studies in epidemiology. [cited 1 Jun 2023]. Available: https://www.strobe-statement.org

44. RCPCH. National Neonatal Audit Programme 2020: Annual Report on 2019 data. 2021. Available: https://www.rcpch.ac.uk/sites/default/files/2021-04/nnap_report_2020-v2.pdf

For peer review only

Figure 1: Overview of the EMNODN NEC care bundle

For peer review only

For peer review only - http://bmjopen.bmj.com/site/about/guidelines.xhtml

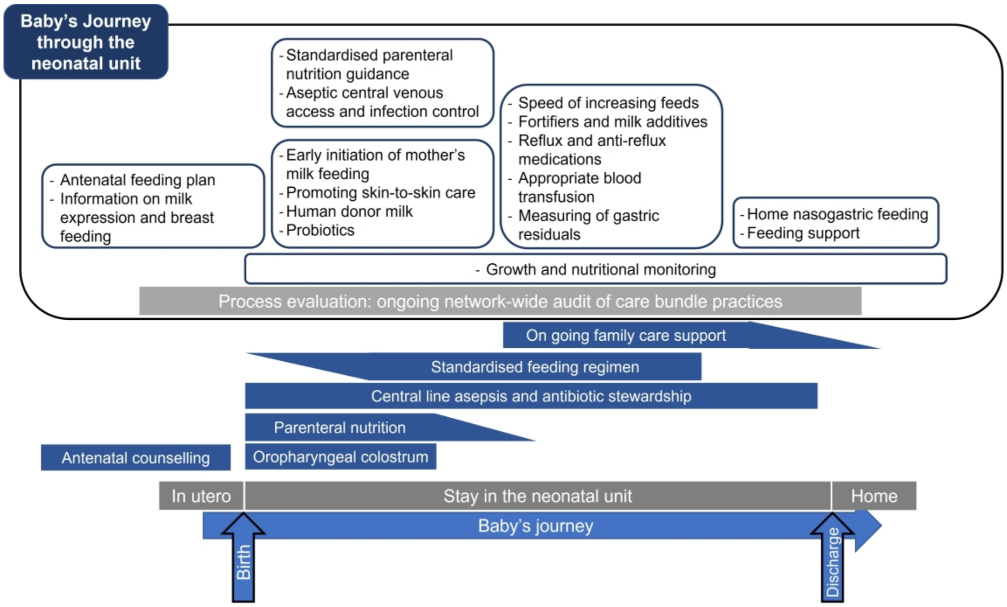

Overview of the EMNODN NEC care bundle

360x217mm (330 x 330 DPI)

BMJ Open

Effects of implementation of a care bundle on rates of necrotising enterocolitis and own mother's milk feeding in the East Midlands: protocol for a mixed methods impact and process evaluation study

Journal:	*BMJ Open*
Manuscript ID	bmjopen-2023-078633.R2
Article Type:	Protocol
Date Submitted by the Author:	20-Feb-2024
Complete List of Authors:	Abramson, Janine; University of Nottingham<br>Szatkowski, Lisa; University of Nottingham, School of Medicine<br>Bains, Manpreet; University of Nottingham, Epidemiology and Public Health<br>Orton, Elizabeth; University of Nottingham<br>Budge, Helen; University of Nottingham; University of Nottingham<br>Spruce, Marie; NEC UK<br>Ojha, Shalini; University of Nottingham; University Hospitals of Derby and Burton NHS Foundation Trust, School of Medicine
**Primary Subject Heading**:	Paediatrics
Secondary Subject Heading:	Evidence based practice, Health services research, Nutrition and metabolism
Keywords:	NUTRITION & DIETETICS, NEONATOLOGY, Nutritional support < GASTROENTEROLOGY

SCHOLARONE™
Manuscripts

Effects of implementation of a care bundle on rates of necrotising enterocolitis and own mother's milk feeding in the East Midlands: protocol for a mixed methods impact and process evaluation study

Janine Abramson[1,2], Lisa Szatkowski[1,2], Manpreet Bains[1], Elizabeth Orton[1], Helen Budge[1,2], Marie Spruce[3], Shalini Ojha[1,2,4]

[1] Lifespan and Population Health, School of Medicine, University of Nottingham, UK

[2] Centre for Perinatal Research, School of Medicine, University of Nottingham, UK

[3] NEC UK, 42 Long Hill Rise, Annesley, Nottingham, NG17 9FG

[4] Neonatal Unit, University Hospitals of Derby and Burton NHS Trust, Derby, UK

Corresponding author: Professor Shalini Ojha, Lifespan and Population Health, School of Medicine, University of Nottingham. Room 4117, Medical School Building, Royal Derby Hospital, Derby DE22 3NE; email: shalini.ojha@nottingham.ac.uk

Orchid ID: https://orcid.org/0000-0001-5668-4227

Key words: infant, preterm; breastfeeding; necrotising enterocolitis

For peer review only

Abstract

Introduction

Prevention of necrotising enterocolitis (NEC) is vital for improving neonatal outcomes. Feeding own mother's milk helps prevent NEC. Rates of own mother's milk feeding in the East Midlands are lower than the national average and the incidence of NEC is higher. The East Midlands Neonatal Operational Delivery Network (EMNODN) has created a care bundle to improve these in babies born at <32 weeks' gestation, the group at the highest risk of NEC. The bundle was introduced in September 2022 and embedded by December 2022. We will evaluate its effectiveness and conduct a process evaluation to understand barriers and facilitators to implementation.

Methods and analysis

We will conduct a retrospective cohort study (Worksteam 1) using data from the National Neonatal Research Database (NNRD). We will identify infants receiving any own mother's milk on day 14 and at discharge, and cases of severe NEC. We will aggregate outcomes by birth-month and use interrupted time series analysis to estimate an incidence rate ratio (IRR) for changes after the care bundle was embedded, relative to pre-implementation. We will model data from all other NNRD units and assess whether there are any concurrent changes to exclude confounding due to other events.

We will apply the RE-AIM framework, (Workstream 2) supplemented by the Consolidated Framework for Implementation Research and Framework for Implementation Fidelity, to conduct a mixed methods evaluation in EMNODN units. We will triangulate data from several sources, including questionnaires and semi-structured interviews with parents and healthcare professionals, and data from patient records.

Ethics and dissemination

For peer review only - http://bmjopen.bmj.com/site/about/guidelines.xhtml

The study has approval from the South East Scotland Research Ethics Committee 01 and the Health Research Authority and Health and Care Research Wales (IRAS 323099). Results will be disseminated via scientific journals and conferences, to neonatal service commissioners and through public-facing infographics.

Study registration

ClinicalTrials.gov NCT05934123

For peer review only

For peer review only - http://bmjopen.bmj.com/site/about/guidelines.xhtml

Article Summary

Strengths and limitations of this study

- Use of data from the National Neonatal Research Database (NNRD) allows comparison of all infants admitted to neonatal units in the East Midlands with a control group of all infants admitted to units elsewhere in England and Wales.
- Interrupted time series analysis will allow any changes in own mother's milk feeding and the incidence of NEC related to implementation of the care bundle to be identified over and above any underlying changing trends.
- The parallel mixed methods process evaluation will allow us to unpack the 'how' and 'why' underpinning the results of the interrupted time series analysis.
- Analysis of routine data will allow all eligible infants to participate without risk of under-recruitment from underserved groups. In the process evaluation, we will purposively seek to include parents from underserved groups who traditionally hesitate to participate in research.
- NEC is a rare occurrence and so statistical power to detect small changes in incidence related to implementation of the care bundle may be limited.

For peer review only

Introduction

Necrotising enterocolitis (NEC) is a serious, life-threatening disease in preterm infants[1]. An estimated 12% of infants born weighing <1500g develop NEC and 30% of these die[2]. Survivors have increased risk of adverse neurodevelopmental outcomes, including cerebral palsy and cognitive impairment[3]. Encouraging and supporting own mother's milk feeding prevents NEC and has several additional advantages, including improved long-term neurodevelopment and parent-infant bonding[4]. Health economic analysis estimates that if all premature infants were to be fed own mother's milk, the total lifetime cost saving to the NHS would be £46.7 million (£30.1 million in the first year of life) and there would be 238 fewer deaths per year with a gain of £153.4 million in lifetime productivity[5].

Neonatal critical care services in England are commissioned by NHS England and managed within 11 Neonatal Operational Delivery Networks (ODNs), to ensure that babies and their families receive high quality, equitable and accessible care. The East Midlands Neonatal Operational Delivery Network (EMNODN[6]) comprises 11 neonatal units across 7 NHS Trusts, covering an area of 15,627km^2, a total population of approximately 4.9 million[7] and approximately 47,000 live births per year[8]. In 2022, EMNODN units provided 50,443 days of care in total to infants born preterm, or unwell at term, who needed specialist neonatal care; 476 infants were cared for who were born before 32 weeks' gestational age and survived at least 48 hours[9].

The rate of NEC in the EMNODN has remained above the national average since 2017, when this measure was added to the National Neonatal Audit Programme, NNAP[10]. Additionally, there are wide variations between the 11 units that comprise the EMNODN. Rates of any breastmilk feeding at discharge from EMNODN units have been consistently below the national average, again with wide local variations.

For peer review only - http://bmjopen.bmj.com/site/about/guidelines.xhtml

The EMNODN has created a NEC care bundle[11] which was introduced to the 11 neonatal units in September 2022, with use fully embedded by December 2022. Care bundles are a group of evidence-based, simple to implement, interventions related to a disease or care process that, when executed together, result in better outcomes than when implemented individually[12]. The EMNODN NEC care bundle consists of recommendations and parent-facing information to promote own mother's milk feeding and optimise nutritional care of preterm infants. Early start and continuation of feeding with own mother's milk such that babies go home breastfeeding is vital to ensure babies continue to receive own mother's milk throughout neonatal care and remain at a reduced risk of NEC.

There is some evidence that quality improvement programmes using care bundles can improve rates of own mother's milk feeding in neonatal units. In the East of England, introduction of a care bundle increased the prevalence of own mother's milk feeding from 50 to 57%[13]. However, no previous studies have explored the effect of complex, multi-component interventions such as a care bundle on rates of NEC and other feeding practices, and there are no published process evaluations of intervention implementation.

Objectives

Our study comprises two parallel, integrated, workstreams with the following objectives:

Workstream 1: Estimate the effects of implementation of the NEC care bundle on rates of own mother's milk feeding and incidence of NEC, comparing infants admitted to the 11 EMNODN neonatal units with admissions to all other units in England and Wales. We will also describe feeding practices before, and after, implementation of the care bundle and examine the infant, maternal and unit-level characteristics associated with own mother's milk feeding and NEC.

Workstream 2: In a multi-centre, mixed methods process evaluation in the 11 EMNODN neonatal units, evaluate how well the care bundle was implemented, understand barriers

For peer review only - http://bmjopen.bmj.com/site/about/guidelines.xhtml

and facilitators to implementation and understand which components contributed most to effectiveness.

Methods and analysis

Design

Parallel, integrated workstreams:

Workstream 1: Retrospective cohort study using routinely collected clinical data, with interrupted time series analysis.

Workstream 2: Multi-centre, mixed methods process evaluation based on the RE-AIM Framework[14,15], Consolidated Framework for Implementation Research (CFIR)[16] and Framework for Implementation Fidelity (FIF)[17].

Setting

Workstream 1: Neonatal units in England and Wales, comparing infants admitted to the 11 neonatal units in the EMNODN with infants admitted to all other units.

Workstream 2: The 11 neonatal units that comprise the EMNODN.

Time period

01 January 2016 to 30 Sept 2023, with infants grouped by date of birth into three groups according to date of implementation of the care bundle: Pre-implementation period – 01 Jan 2016 to 31 Aug 2022; Implementation period – 01 Sep 2022 to 31 Dec 2022; Evaluation period – 01 Jan 2023 to 30 Sep 2023, followed up for 3 months (until 31 Dec 2023) to cover the full neonatal stay of the final participant.

Data source

Workstream 1: Routinely recorded electronic patient record data from all admissions to NHS neonatal units in England and Wales, held within the National Neonatal Research Database (NNRD). Data are entered by clinical staff at the point of care and extracted, anonymised

For peer review only - http://bmjopen.bmj.com/site/about/guidelines.xhtml

and combined by the Neonatal Data Analysis Unit (NDAU) at Imperial College, London. The NNRD has NHS Research Ethics Committee approval (10/80803/151) and approval from the Caldicott Guardians of NHS Trusts for research use.

Workstream 2: Quantitative and qualitative data from a variety of sources: questionnaires with parents and HCPs; semi-structured one-to-one interviews with parents and HCPs; NNRD data, supplemented with data directly from paper-based and electronic patient notes, collected using an electronic case report form, for data items not available within the NNRD; data from EMNODN units on cot capacity, staffing levels and facilities.

Questionnaires will be completed electronically using an online survey platform, with paper copies available should participants request them. Any data from questionnaires completed on paper will be manually digitised, with a sample (10%) checked for verification of data entry. Audio recordings of interviews will be transcribed verbatim and transcripts will be checked against audio recordings to ensure they are accurate and that any identifiable information discussed during interviews is anonymised.

Eligibility criteria

For Workstream 1, we will include infants who: were born at <32 weeks gestational age (GA); survived at least 48 hours from birth; were admitted for neonatal care from 01 January 2016 to 30 September 2023 to a neonatal unit contributing data to the NNRD. In line with previous work[18,19], we will exclude infants with missing data on key characteristics (e.g., sex, gestational age, final discharge destination) as well as infants admitted >12 hours after birth and those missing data for 1 or more episodes of care.

For Workstream 2, we will include parents of infants who are: admitted to one of the 11 EMNODN neonatal units in a 6-month period after implementation of the care bundle; born at <32 weeks GA; survived at least 48 hours from birth. All eligible parents will be invited to respond to the questionnaires. For the interviews, parents will be purposively sampled to

For peer review only - http://bmjopen.bmj.com/site/about/guidelines.xhtml

include: parents from different neonatal units; parents of extremely preterm infants (born at 22-27 weeks GA) and very preterm infants (born at 28-32 weeks GA); parents whose infants received own mother's milk, mixed feeding, or did not receive any own mothers' milk; parents of infants who had suspected or confirmed NEC; parents representing different maternal (e.g., education) and environmental (including socioeconomic) factors.

We will recruit HCPs working in EMNODN neonatal units. All HCPs will be invited to respond to the questionnaires and some will be purposely sampled to include a range of individuals representing different job roles (unit leads, consultants, junior doctors, nurse practitioners, nurses, health care assistants and allied health professionals including dietitians, speech and language therapists) and hospital sites.

All questionnaire respondents and interview participants will provide written informed consent for their participation and use of the data provided, including deidentified direct quotations in study outputs.

Intervention

The NEC care bundle, designed by a multi-disciplinary team, consists of recommendations for neonatal units, alongside parent-facing information, to promote own mother's milk feeding and ensure optimum nutritional care of preterm infants. A schematic representation of the care bundle is shown in Figure 1, giving an overview of an infant's journey through neonatal care and opportunities to reduce the risk of NEC.

Recommendations to optimise nutritional care include guidance on: ensuring early and adequate parenteral nutrition given when infants cannot have sufficient milk feeds such as in the first hours after birth, while building milk feeds and when unwell (following established NICE guidelines[20]); use of oropharyngeal colostrum[21] (the practice of giving own mothers' first milk directly into the infant's oropharynx to enhance immunity and protect against NEC); optimal time and speed of increasing milk feeds[22]; and use of other interventions such as human donor milk[23], probiotics[24], and breast milk fortifiers[25].

For peer review only - http://bmjopen.bmj.com/site/about/guidelines.xhtml

For peer review only

Primary outcomes

- Receiving any own mother's milk at discharge (exclusively or in combination with infant formula).
- Receiving any own mother's milk on day 14 of life (exclusively or in combination with infant formula).
- Severe NEC requiring surgery or resulting in death[26].

Secondary outcomes

- Exclusive feeding with own mother's milk at discharge.
- Any NEC, including NEC not requiring surgery and not resulting in death.

Data analysis strategy: Workstream 1

After data cleaning, the number of infants with missing data across key variables will be tabulated and the total number of exclusions and final study population size determined. Descriptive statistics will be presented to summarise the distribution of baseline variables during each of the three study phases, separately for the EMNODN units (intervention group) and all other units in England and Wales (control group). Continuous variables will be reported with means and standard deviations, if shown to be normally distributed using a combined skewness and kurtosis test, or otherwise will be reported using medians and interquartile ranges (IQR). Categorical variables will be reported with frequencies and percentages. P-values for differences between the three time periods will be reported, using the Chi squared test or the Wilcoxon rank-sum test, as appropriate.

We will use interrupted time series (ITS) analysis to assess the impact of implementation of the care bundle on the outcomes listed above, as has been used previously in neonatal research[27]. This is a strong quasi-experimental design which accounts for underlying trends in an outcome. If, for example, rates of NEC were already in decline prior to the

For peer review only - http://bmjopen.bmj.com/site/about/guidelines.xhtml

For peer review only

implementation of the bundle, the ITS methodology will detect any additional impact of the bundle, above the background decline. It can also account for the impact of any other interventions or changes in population characteristics which might themselves impact outcomes.

We will aggregate outcomes by month of birth (or by two-month period if necessary to improve statistical power, without detriment to interpretation of results) and plot time series graphs to show the number of cases of each outcome per month over the study period in the EMNODN units combined. We will use a quasi-Poisson generalised additive mixed model[28] (GAMM) to estimate an incidence rate ratio (IRR) for changes in the incidence of the outcomes in EMNODN units during the evaluation phase, relative to the pre-implementation period. Data for the 4-month implementation phase will be plotted graphically but excluded from statistical modelling as this period is too short to be able to model changes in incidence compared to the pre-implementation period. We will similarly plot and model data for infants admitted to units in the control group, to assess whether there are any changes in the IRR to exclude confounding due to other events or interventions which occurred around the time that the care bundle was introduced.

We will incorporate several covariates into our models as follows:

- The total number of admissions per month will be used as an offset term in each model to account for variations in the number of babies ‘at risk’ of the outcomes.
- The secular trend in each outcome will be captured using a continuous variable coded from 1 to 93 (the total number of months of data across the study period).
- To assess whether there was a step change in the outcomes, we will include a binary variable coded as 0 during the pre-implementation phase and 1 during the evaluation phase.
- To assess whether there was any change in trend (upwards or downwards) in the outcomes, we will include a binary variable coded as 0 during the pre-implementation

For peer review only - http://bmjopen.bmj.com/site/about/guidelines.xhtml

phase and from 1 to 9 (the number of months in the evaluation phase) during the evaluation phase. We will also assess whether there are any quadratic or cubic trends in the outcomes in the evaluation period, though the relatively short (9 month) length of this evaluation period will limit the ability to detect any non-linear trends. We will use the Akaike Information Criterion (AIC) to compare models with linear, quadratic and cubic trends in the evaluation period, with a smaller AIC indicating better model fit.

- Month of the year will be included as a cyclic cubic spline to capture any non-linear, seasonal patterns in outcomes, such as the impact of activities related to the annual World NEC Awareness Day[29] and International Kangaroo Care Awareness Day[30] (both held annually in May), or any variation in NEC incidence related to seasonality of circulating pathogens[31].
- To account for any potential impact of the COVID-19 pandemic, we will include a binary variable coded as 1 during periods of national 'lockdown' and 0 otherwise, in line with our previous work examining the impact of pandemic-related visitor restrictions on own mother's milk feeding[32].
- ITS studies are not usually affected by confounders relating to population characteristics as these do not typically change significantly over time[33]. If the characteristics of infants born during each of the three study time periods differ, we will include covariates to capture any differences in potential confounders by month of birth, such as: median GA; percentage of admissions male; median birthweight; percentage of infants with a high-risk NMR-2000 score (a validated score of in-hospital mortality risk)[34]; percentage of infants with a major congenital anomaly; median length of stay.
- Plots of the autocorrelation function (ACF) and partial autocorrelation function (PACF) will be examined to identify plausible values of the autoregressive (AR) and/or moving average (MA) terms to capture autocorrelation between data points.

For peer review only - http://bmjopen.bmj.com/site/about/guidelines.xhtml

We will use the Akaike Information Criterion (AIC) to compare models with different plausible values of AR and MA terms, with a smaller AIC indicating better model fit.

Multiple testing: IRRs for the primary and secondary outcomes will be reported with 95% confidence intervals and Bonferroni-corrected p-values.

Secondary analyses: In addition to the ITS analysis, we will use multivariable logistic regression to conduct a conventional before-after comparison of the odds of each primary and secondary outcome, adjusted for a range of potential confounders measured at the level of the infant, mother and unit. We will include data from both EMNODN and control units and assess whether there is any effect modification by study group. We will use a robust variance estimator to account for the clustering of infants within units and by mother.

Missing data: We will describe the amount of missing data for covariates. For the multivariable logistic regression, we will assume that any missing data occur randomly between the study phases and intervention and control groups and will impute missing values 10-fold using chained equations. Results will be pooled according to Rubin's rule.

Data analysis strategy: Workstream 2

We will use the RE-AIM framework[14,15], supplemented by the Consolidated Framework for Implementation Research (CFIR)[16] and the Framework for Intervention Fidelity (FIF)[17], to examine implementation of the care bundle, mechanisms of impact and the impact of contextual factors, to map the 'how' and 'why' underpinning the results of the quantitative evaluation undertaken in Workstream 1. These frameworks bring together metrics for evaluating implementation success, and by assessing reach and effectiveness the frameworks can be used together to identify ways to produce sustained changes (RE-AIM and FIF), which will highlight modifiable factors that explain and enhance implementation outcomes (CFIR). We will triangulate data from the NNRD, case report

For peer review only - http://bmjopen.bmj.com/site/about/guidelines.xhtml

forms, and parent and HCP questionnaires and interviews to address the five evaluative dimensions of RE-AIM and map the CFIR constructs and FIF checklist within these domains (as appropriate e.g., particularly around Adoption, Implementation and Maintenance), to understand factors impacting implementation:

1) Reach: we will present information on numbers and percentages of infants and parents exposed to different elements of the care bundle and any exceptions or exclusions, considering variation by socio-economic and demographic characteristics. We will measure numbers of HCPs trained to deliver the care bundle and perceptions of the acceptability and usefulness of training.
2) Efficacy: in addition to the overall efficacy assessment in Workstream 1, we will measure the efficacy of key components of the care bundle and investigate their impact on individual level outcomes. For example, we will compare rates of own mother's milk feeding between mothers who received antenatal breastfeeding counselling and mothers who did not.
3) Adoption: with HCPs and unit leads we will explore the contextual factors that vary between units that may impact adoption and fidelity of recommended practices, using the FIF checklist and CFIR to guide thematic coding. Moderators of adoption and fidelity may include staff training and existing expertise, level of available resource locally.
4) Implementation: we will explore factors that may affect implementation of the care bundle, using CFIR as a coding framework. This will include factors related to the care bundle (e.g., its complexity and evidence-base), factors related to setting (e.g., staff capacity to deliver the care bundle, availability of resources, time and expertise, and leadership buy-in), attributes of roles within the teams and their motivations to implement the care bundle, and if and how the care bundle is adapted in each setting. This will help us better understand barriers and facilitators to implementation of the bundle.
5) Maintenance: we will assess the extent to which the care bundle is embedded in training, policy and guidance.

For peer review only - http://bmjopen.bmj.com/site/about/guidelines.xhtml

Interview data will be analysed using the framework approach[35,36], a hierarchical, matrix-based method to map thematic differences/similarities within and between groups. First, a priori codes relating to the RE-AIM domains (Adoption, Implementation and Maintenance) will be applied to the data. Transcripts will then be coded again to apply the CFIR domains, constructs or subconstructs and FIF checklist (identified to influence RE-AIM domains), inductively. As an initial step to check validity of coding, two researchers will compare coding of several transcripts to reach consensus on interpretations. The remaining data will then be coded according to the relevant RE-AIM, CFIR and FIF code domains and construct codes, and the generated themes, with matrices of results and quotes presented and discussed among the full research team, to agree on data interpretation and the final themes.

Sample size calculation

Workstream 1: The interrupted time series analysis will have approximately 90% power to detect an absolute increase of 20% in the prevalence of receiving own mother's milk at discharge and 80% power to detect a 15% absolute increase (baseline prevalence c.55%, c.23 infants per month in the EMNODN from 2017-19; national average c.60%). This target is comparable to the prevalence of own mother's milk feeding in the best performing neonatal network - in 2021, the South London Neonatal ODN achieved a prevalence of 76% (range by unit 71% to 80%) of infants receiving own mother's milk at discharge[37]. In 21 neonatal units in the UK in 2021, over 75% of infants received own mother's milk at discharge, with the highest performing unit achieving 86% prevalence[37].

NEC is a rare occurrence (incidence c.7%, c.3 cases per month in the EMNODN from 2017-19; national average c.5%) and there is also considerable monthly variability in incidence. Therefore, statistical power to detect small changes in the incidence of NEC will be more limited; we will be powered to detect approximately a two-thirds reduction in the incidence of NEC. In line with previous studies of less common neonatal outcomes[27], the point estimate

For peer review only - http://bmjopen.bmj.com/site/about/guidelines.xhtml

will, however, give an indication of the effect of the care bundle, even if the confidence interval around this point estimate crosses the null value.

Workstream 2: We aim to conduct 33 interviews with parents (either individuals or couples, 3 per EMNODN unit) and 33 interviews with HCPs (3 per unit) using a combination of convenience (self-selecting) and purposeful sampling approaches, the latter particularly to approach parents from under-served groups who usually hesitate to participate in research. These numbers are based on principles of maximum variation and theoretical data saturation, which is related to the sampling approach rather than the analysis[38]. Preliminary analysis will inform whether we need to use a second strategy based on the notion of inductive thematic saturation[39] and, in which case, we may need to conduct additional interviews.

Patient and Public Involvement

The study protocol has been co-produced with the EMNODN parent advisory group (PAG) and the parent-led charity NEC UK[40] to ensure representation from those with lived experiences of NEC and its consequences, both in the neonatal unit and after discharge. The chair of NEC UK is a parent co-applicant and will lead PPI activities. A second PPI representative will also be invited to join the Project Management Group.

The primary outcome of receiving own mother's milk feeds at discharge was selected by the parents as being the most meaningful outcome for them, and, further to their advice, the emphasis on receiving information and support for breast milk expression in the process evaluation and the plans to explore this in detail in the semi-structured parent interviews were included in the study plan.

For peer review only - http://bmjopen.bmj.com/site/about/guidelines.xhtml

With the support of The Centre for Ethnic Health Research[41], a semi-structured focus group discussion has been conducted with women representing different ethnic minority groups, all of whom had experience of having a preterm baby. This discussion focused on co-production of study documentation, particularly the participant information sheets and interview guides. A semi-structured focus group discussion has also been conducted with HCPs working with preterm infants in the East Midlands. This discussion focused on the production of the interview guide for HCPs. Interested parties from both focus groups will be invited to validate qualitative analyses (respondent validation), comment on results and recommendations and review and revise the public-facing dissemination materials. A final parent-led dissemination meeting will be held within the EMNODN.

Ethics and dissemination

The study is sponsored by the University Hospitals of Derby and Burton NHS Foundation Trust and has been registered on the ClinicalTrials.gov database (Ref: NCT05934123). The study has been approved by the South East Scotland Research Ethics Committee 01 and the Health Research Authority and Health and Care Research Wales (IRAS 323099).

We will use the RECORD checklist[42], an extension of the STROBE guidelines[43], to ensure transparency of reporting of our methods and results. On completion of the study, findings will be disseminated via: a detailed report for NIHR as well as the EMNODN and participating neonatal units; public facing materials (infographics, animated video) in English, Polish, Gujarati, and Punjabi (the most frequently-spoken languages in the East Midlands), created and shared via parent groups and directly with study participants; open access, peer-reviewed journals and scientific conferences; and dissemination to relevant specialised commissioners including the NHS England Neonatal Critical Care Clinical Reference Group.

For peer review only

Discussion

National recommendations cite a need for quality improvement (QI) efforts to increase own mother's milk feeding to reduce NEC[44]. In 2020, NNAP recommended that if rates of NEC are relatively high, neonatal networks and units should "seek to identify and implement potentially better practices" and to "focus on the early initiation and sustainment of breastmilk feeding in conjunction with parents by reviewing data and processes" and "undertake selected QI activities suited to the local context"[44]. This multi-component evaluation of the care bundle developed for use in the East Midlands will provide evidence to help understand whether the bundle should be rolled out nationally and what changes might be needed to ensure optimum impact and successful implementation within local contexts. Thus, the findings of this study have the potential to improve patient care in the EMNODN and wider, with direct benefits to preterm infants and their families. The parallel effectiveness and process evaluations will establish a framework and toolkit for evaluation of other similar QI initiatives in future.

Patient consent for publication: Not applicable

Acknowledgements

We acknowledge the East Midlands Neonatal Operational Delivery Network team for their support in setting up the study. We also acknowledge the authors of the EMNODN Care Bundle (Dr Dushyant Batra, Dr Deepa Panjwani, Dr Lara Shipley, Dr Chiara Taylor, Dr Rizma Aishath Moosa, and Ms. Ruth Prigg) for their contribution to the creation of the care bundle.

Electronic patient data recorded at participating neonatal units that collectively form the United Kingdom Neonatal Collaborative are transmitted to the Neonatal Data Analysis Unit to form the National Neonatal Research Database (NNRD). We are grateful to all the

For peer review only

families that agreed to the inclusion of their baby's data in the NNRD, the health professionals who recorded data and the Neonatal Data Analysis Unit team.

Author contributions

SO conceptualised and designed the study, contributed to analyses plan, and will lead the delivery and reporting. LS planned the statistical analysis and MB and EO planned the mixed methods process evaluation. JA is the study manager and will perform the qualitative data collection and analysis. HB contributed to study design and will provide input into the interpretation and writing of the results. MS is the PPI Lead and will lead development of public-facing dissemination materials. All authors contributed to the overall study design, development of the protocol and the writing and review of this paper.

Data Statement

Technical appendix, statistical codes, and dataset will be available from the corresponding author.

Funding statement

This project is funded by the National Institute for Health and Care Research (NIHR) under its Research for Patient Benefit (RfPB) Programme (Grant Reference Number NIHR203590). The views expressed are those of the author(s) and not necessarily those of the NIHR or the Department of Health and Social Care.

Competing interests statement

For peer review only

SO is an Honorary Neonatal Consultant at the University Hospitals of Derby and Burton and is the lead clinician in the team that developed the EMNODN NEC Care Bundle. HB is an Honorary Neonatal Consultant at the Nottingham University Hospitals and was on the advisory panel of the team that developed the EMNODN NEC Care Bundle. MS is the chair of NEC-UK which supports families with experience of NEC and supports breastfeeding mothers. LS, MB, JA and EO have no conflicts of interest to declare.

For peer review only

Word Count: 3,777 words

References

1. Rose AT, Patel RM. A critical analysis of risk factors for necrotizing enterocolitis. Semin Fetal Neonatal Med. 2018;23: 374–379. doi:10.1016/j.siny.2018.07.005

2. Battersby C, Longford N, Mandalia S, Costeloe K, Modi N, UK Neonatal Collaborative Necrotising Enterocolitis (UKNC-NEC) study group. Incidence and enteral feed antecedents of severe neonatal necrotising enterocolitis across neonatal networks in England, 2012-13: a whole-population surveillance study. Lancet Gastroenterol Hepatol. 2017;2: 43–51. doi:10.1016/S2468-1253(16)30117-0

3. Adams-Chapman I. Necrotizing Enterocolitis and Neurodevelopmental Outcome. Clin Perinatol. 2018;45: 453–466. doi:10.1016/j.clp.2018.05.014

4. Victora CG, Bahl R, Barros AJD, França GVA, Horton S, Krasevec J, et al. Breastfeeding in the 21st century: epidemiology, mechanisms, and lifelong effect. The Lancet. 2016;387: 475–490. doi:10.1016/S0140-6736(15)01024-7

5. Mahon J, Claxton L, Wood H. Modelling the cost-effectiveness of human milk and breastfeeding in preterm infants in the United Kingdom. Health Econ Rev. 2016;6: 54. doi:10.1186/s13561-016-0136-0

6. EMNODN. East Midlands Neonatal Operational Delivery Network. [cited 20 Apr 2023]. Available: https://www.emnodn.nhs.uk

7. Office for National Statistics. 2021 Census Profile for areas in England and Wales: East Midlands Region. [cited 10 Jan 2024]. Available: https://www.nomisweb.co.uk/sources/census_2021/report?compare=E12000004

8. Office for National Statistics. Births in England and Wales: 2022. [cited 11 Jan 2024]. Available:

For peer review only - http://bmjopen.bmj.com/site/about/guidelines.xhtml

https://www.ons.gov.uk/peoplepopulationandcommunity/birthsdeathsandmarriages/livebirths/bulletins/birthsummarytablesenglandandwales/2022

9. Royal College of Paediatrics and Child Health. Neonatal unit and neonatal network annual summary reports. [cited 10 Dec 2023]. Available: https://nnap.rcpch.ac.uk/annual-reports.aspx

10. Royal College of Paediatrics and Child Health. National Neonatal Audit Programme. [cited 20 Apr 2023]. Available: https://www.rcpch.ac.uk/work-we-do/clinical-audits/nnap

11. East Midlands Neonatal Operational Delivery Network. Network Guideline: Necrotising enterocolitis care bundle. 2022. Available: https://www.emnodn.nhs.uk/_files/ugd/143840_5050aa59d39b4c66bf2582d7491ba478.pdf

12. Resar R, Griffin F, Haraden C, Nolan T. Using Care Bundles to Improve Health Care Quality. Cambridge, Massachusetts: Institute for Healthcare Improvement; 2021. Available: http://www.ihi.org/resources/Pages/IHIWhitePapers/UsingCareBundles.aspx

13. Battersby C, Santhakumaran S, Upton M, Radbone L, Birch J, Modi N, et al. The impact of a regional care bundle on maternal breast milk use in preterm infants: outcomes of the East of England quality improvement programme. Arch Dis Child Fetal Neonatal Ed. 2014;99: F395-401. doi:10.1136/archdischild-2013-305475

14. Skivington K, Matthews L, Simpson SA, Craig P, Baird J, Blazeby JM, et al. A new framework for developing and evaluating complex interventions: update of Medical Research Council guidance. BMJ. 2021; n2061. doi:10.1136/bmj.n2061

15. Glasgow RE, Harden SM, Gaglio B, Rabin B, Smith ML, Porter GC, et al. RE-AIM Planning and Evaluation Framework: Adapting to New Science and Practice With a 20-Year Review. Front Public Health. 2019;7: 64. doi:10.3389/fpubh.2019.00064

16. Damschroder LJ, Reardon CM, Widerquist MAO, Lowery J. The updated Consolidated Framework for Implementation Research based on user feedback. Implement Sci. 2022;17: 75. doi:10.1186/s13012-022-01245-0

17. Carroll C, Patterson M, Wood S, Booth A, Rick J, Balain S. A conceptual framework for implementation fidelity. Implement Sci IS. 2007;2: 40. doi:10.1186/1748-5908-2-40

18. Sand L, Szatkowski L, Kwok TC, Sharkey D, Todd DA, Budge H, et al. Observational cohort study of changing trends in non-invasive ventilation in very preterm infants and associations with clinical outcomes. Arch Dis Child Fetal Neonatal Ed. 2021; fetalneonatal-2021-322390. doi:10.1136/archdischild-2021-322390

19. Szatkowski L, Fateh S, Abramson J, Kwok TC, Sharkey D, Budge H, et al. Observational cohort study of use of caffeine in preterm infants and association between early caffeine use and neonatal outcomes. Arch Dis Child Fetal Neonatal Ed. 2023; fetalneonatal-2022-324919. doi:10.1136/archdischild-2022-324919

20. NICE. Neonatal parenteral nutrition NICE guideline [NG154]. 2020. Available: https://www.nice.org.uk/guidance/ng154

21. Nasuf AWA, Ojha S, Dorling J. Oropharyngeal colostrum in preventing mortality and morbidity in preterm infants. Cochrane Neonatal Group, editor. Cochrane Database Syst Rev. 2018;2019. doi:10.1002/14651858.CD011921.pub2

For peer review only

22. Dorling J, Abbott J, Berrington J, Bosiak B, Bowler U, Boyle E, et al. Controlled Trial of Two Incremental Milk-Feeding Rates in Preterm Infants. N Engl J Med. 2019;381: 1434–1443. doi:10.1056/NEJMoa1816654

23. Quigley M, Embleton ND, McGuire W. Formula versus donor breast milk for feeding preterm or low birth weight infants. Cochrane Neonatal Group, editor. Cochrane Database Syst Rev. 2019 [cited 17 Nov 2021]. doi:10.1002/14651858.CD002971.pub5

24. Sharif S, Meader N, Oddie SJ, Rojas-Reyes MX, McGuire W. Probiotics to prevent necrotising enterocolitis in very preterm or very low birth weight infants. Cochrane Neonatal Group, editor. Cochrane Database Syst Rev. 2020;2020. doi:10.1002/14651858.CD005496.pub5

25. Brown JVE, Lin L, Embleton ND, Harding JE, McGuire W. Multi-nutrient fortification of human milk for preterm infants. Cochrane Neonatal Group, editor. Cochrane Database Syst Rev. 2020;2020. doi:10.1002/14651858.CD000343.pub4

26. Battersby C, Santhalingam T, Costeloe K, Modi N. Incidence of neonatal necrotising enterocolitis in high-income countries: a systematic review. Arch Dis Child - Fetal Neonatal Ed. 2018;103: F182–F189. doi:10.1136/archdischild-2017-313880

27. Szatkowski L, McClaughry R, Clarkson M, Sharkey D. Restricted visiting reduces nosocomial viral respiratory tract infections in high-risk neonates. Eur Respir J. 2019;53: 1801874. doi:10.1183/13993003.01874-2018

28. Wood SN. Fast Stable Restricted Maximum Likelihood and Marginal Likelihood Estimation of Semiparametric Generalized Linear Models. J R Stat Soc Ser B Stat Methodol. 2011;73: 3–36. doi:10.1111/j.1467-9868.2010.00749.x

29. NEC UK. NEC Awareness Day. [cited 12 May 2023]. Available: https://www.necuk.org.uk/nec-awareness-day

30. Kangaroo Mother Care. Kangaroo Care Awareness Day. [cited 12 May 2023]. Available: https://kangaroo.care

31. Snyder CL, Hall M, Sharma V, St. Peter SD. Seasonal variation in the incidence of necrotizing enterocolitis. Pediatr Surg Int. 2010;26: 895–898. doi:10.1007/s00383-010-2675-5

32. Abdul Hamid HB, Szatkowski L, Budge H, Ojha S. Impact of the coronavirus disease 2019 pandemic on breastfeeding during and at discharge from neonatal care: An observational cohort study. Pediatr Investig. 2022;6: 189–196. doi:10.1002/ped4.12337

33. Bernal JL, Cummins S, Gasparrini A. Interrupted time series regression for the evaluation of public health interventions: a tutorial. Int J Epidemiol. 2017;46: 348–355. doi:10.1093/ije/dyw098

34. Medvedev MM, Brotherton H, Gai A, Tann C, Gale C, Waiswa P, et al. Development and validation of a simplified score to predict neonatal mortality risk among neonates weighing 2000 g or less (NMR-2000): an analysis using data from the UK and The Gambia. Lancet Child Adolesc Health. 2020;4: 299–311. doi:10.1016/S2352-4642(20)30021-3

35. Ritchie J, Lewis J, Elam G. Qualitative Research Practice: A Guide for Social Science Students and Researchers. London: Sage; 2003.

For peer review only - http://bmjopen.bmj.com/site/about/guidelines.xhtml

36. Gale NK, Heath G, Cameron E, Rashid S, Redwood S. Using the framework method for the analysis of qualitative data in multi-disciplinary health research. BMC Med Res Methodol. 2013;13: 117. doi:10.1186/1471-2288-13-117

37. Royal College of Paediatrics and Child Health. National Neonatal Audit Programme Summary report on 2021 data. 2023. Available: https://www.rcpch.ac.uk/resources/national-neonatal-audit-programme-summary-report-2021-data

38. Emmel N. Sampling and Choosing Cases in Qualitative Research: A Realist Approach. 1 Oliver's Yard, 55 City Road, London EC1Y 1SP United Kingdom: SAGE Publications Ltd; 2013. doi:10.4135/9781473913882

39. Saunders B, Sim J, Kingstone T, Baker S, Waterfield J, Bartlam B, et al. Saturation in qualitative research: exploring its conceptualization and operationalization. Qual Quant. 2018;52: 1893–1907. doi:10.1007/s11135-017-0574-8

40. NEC UK. [cited 30 Apr 2023]. Available: https://www.necuk.org.uk

41. The Centre for Ethnic Health Research. [cited 30 Apr 2023]. Available: https://ethnichealthresearch.org.uk

42. RECORD: REporting of studies Conducted using Observational Routinely-collected Data. [cited 1 Jun 2023]. Available: https://www.record-statement.org

43. STROBE: Strengthening the reporting of observational studies in epidemiology. [cited 1 Jun 2023]. Available: https://www.strobe-statement.org

44. RCPCH. National Neonatal Audit Programme 2020: Annual Report on 2019 data. 2021. Available: https://www.rcpch.ac.uk/sites/default/files/2021-04/nnap_report_2020-v2.pdf

For peer review only

For peer review only - http://bmjopen.bmj.com/site/about/guidelines.xhtml

Figure 1: Overview of the EMNODN NEC care bundle

For peer review only

For peer review only - http://bmjopen.bmj.com/site/about/guidelines.xhtml

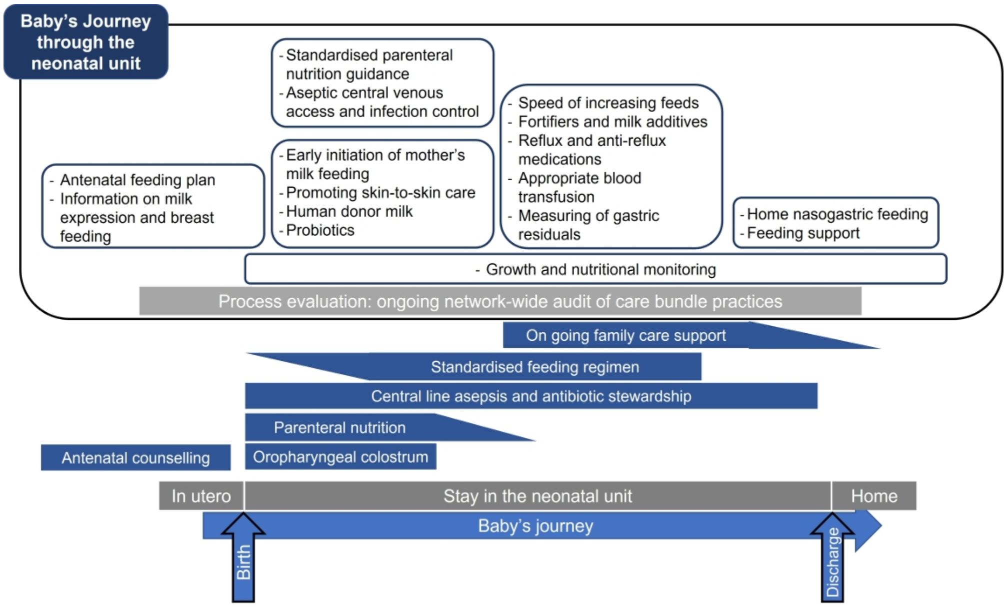

Overview of the EMNODN NEC care bundle

360x217mm (330 x 330 DPI)
